# Comparison of Methods to Account for Relatedness in Genome-Wide Association Studies with Family-Based Data

**DOI:** 10.1371/journal.pgen.1004445

**Published:** 2014-07-17

**Authors:** Jakris Eu-ahsunthornwattana, E. Nancy Miller, Michaela Fakiola, Wellcome Trust Case Control Consortium 2, Selma M. B. Jeronimo, Jenefer M. Blackwell, Heather J. Cordell

**Affiliations:** 1 Institute of Genetic Medicine, Newcastle University, International Centre for Life, Newcastle upon Tyne, United Kingdom; 2 Division of Medical Genetics, Department of Internal Medicine, Faculty of Medicine Ramathibodi Hospital, Mahidol University, Ratchathevi, Bangkok, Thailand; 3 Cambridge Institute for Medical Research, University of Cambridge School of Clinical Medicine, Addenbrooke's Hospital, Cambridge, United Kingdom; 4 Department of Biochemistry, Center for Biosciences, Universidade Federal do Rio Grande do Norte, Natal, Brazil; 5 Telethon Institute for Child Health Research, Centre for Child Health Research, The University of Western Australia, Subiaco, Western Australia, Australia; University of Michigan, United States of America

## Abstract

Approaches based on linear mixed models (LMMs) have recently gained popularity for modelling population substructure and relatedness in genome-wide association studies. In the last few years, a bewildering variety of different LMM methods/software packages have been developed, but it is not always clear how (or indeed whether) any newly-proposed method differs from previously-proposed implementations. Here we compare the performance of several LMM approaches (and software implementations, including EMMAX, GenABEL, FaST-LMM, Mendel, GEMMA and MMM) via their application to a genome-wide association study of visceral leishmaniasis in 348 Brazilian families comprising 3626 individuals (1972 genotyped). The implementations differ in precise details of methodology implemented and through various user-chosen options such as the method and number of SNPs used to estimate the kinship (relatedness) matrix. We investigate sensitivity to these choices and the success (or otherwise) of the approaches in controlling the overall genome-wide error-rate for both real and simulated phenotypes. We compare the LMM results to those obtained using traditional family-based association tests (based on transmission of alleles within pedigrees) and to alternative approaches implemented in the software packages MQLS, ROADTRIPS and MASTOR. We find strong concordance between the results from different LMM approaches, and all are successful in controlling the genome-wide error rate (except for some approaches when applied naively to longitudinal data with many repeated measures). We also find high correlation between LMMs and alternative approaches (apart from transmission-based approaches when applied to SNPs with small or non-existent effects). We conclude that LMM approaches perform well in comparison to competing approaches. Given their strong concordance, in most applications, the choice of precise LMM implementation cannot be based on power/type I error considerations but must instead be based on considerations such as speed and ease-of-use.

## Author Summary

Recently, statistical approaches known as linear mixed models (LMMs) have become popular for analysing data from genome-wide association studies. In the last few years, a bewildering variety of different LMM methods/software packages have been developed, but it has not always been clear how (or indeed whether) any newly-proposed method differs from previously-proposed implementations. Here we compare the performance of several different LMM approaches (and software implementations) via their application to a genome-wide association study of visceral leishmaniasis in 348 Brazilian families comprising 3626 individuals. We also compare the LMM results to those obtained using alternative analysis methods. Overall, we find strong concordance between the results from the different LMM approaches and high correlation between the results from LMMs and most alternative approaches. We conclude that LMM approaches perform well in comparison to competing approaches and, in most applications, the precise LMM implementation will not be too important, and can be chosen on the basis of speed or convenience.

## Introduction

Recently, linear mixed models based approaches have been proposed as appealing alternatives to principal component based approaches when adjusting for population substructure in genome-wide association studies of apparently unrelated individuals [1]–[4]. These methods build upon work originally described in the animal breeding literature, and subsequently developed in the human genetics literature, in which a genetic effect of interest (e.g. the number of copies of a particular allele at a particular test SNP) is included as a fixed effect in a regression model, with an additional random effect also included to model genetic correlation between individuals. The covariance structure for the random effect is generally assumed to correspond to that implied by a polygenic model, incorporating the genetic relationship (kinship) between each pair of individuals. Although use of this linear mixed model (LMM) was originally proposed for pedigrees with known relationships [5]–[10], this approach has recently gained popularity for use with samples of unknown or uncertain relationship [1]–[3], [11]–[13], including apparently unrelated samples who may nevertheless display distant levels of common ancestry. For this purpose, the kinship coefficients between all pairs of individuals modelling either close or distant relatedness are estimated (prior to fitting the linear mixed model) on the basis of genome-wide genotype data, rather than being fixed at their known theoretical values.

Fitting a full linear mixed model for each SNP in turn across the genome is computationally challenging. These computational considerations have led to the development of several faster approximations for constructing tests of the fixed SNP effects of interest in the linear mixed model [1], [2], [9], [10], [14]. These approximate tests have been implemented in various software packages including MERLIN, GenABEL, EMMAX, TASSEL, FaST-LMM, Mendel and MMM. The MMM [15] and FaST-LMM [4] packages, in common with the package GEMMA [16], also provide fast implementations of an exact (rather than an approximate) model, which in principle can lead to a small increase in power [15], [16], depending on the true underlying level of relatedness.

A limited comparison of several LMM implementations, via application to real and simulated data from Genetic Analysis Workshop 18 (GAW18) [17], was performed by Eu-ahsunthornwattana et al. [18]. In the GAW18 data, which comprised 959 Mexican-American individuals from 20 families, the LMM implementations investigated performed rather similarly to one another in terms of the association test statistics and p-values achieved; however, no formal quantification of power or type 1 error was performed. Eu-ahsunthornwattana et al. [18] also investigated the performance of the various LMM implementations when applied naively to longitudinal traits (repeated measures) available in GAW18, simply by treating each measurement as if it came from a separate person and expanding out the genetic data set accordingly (resulting in an expanded data set containing many apparent twins, triplets, quadruplets etc., depending on how many measurements are available for each person). Although this approach is not strictly ‘correct’ (as it does not distinguish between correlations in trait values due to genetic factors and correlations due to non-genetic within-individual factors), Eu-ahsunthornwattana et al. found this procedure generated only minimal inflation in the resulting distribution of genome-wide test statistics.

Here we expand the investigation of Eu-ahsunthornwattana et al. [18] to perform a more comprehensive comparison of LMM approaches (involving a larger number of software implementations) and to conduct a formal investigation of power and type 1 error. We also compare the LMM approaches to traditional family-based approaches (‘within-family association tests’ based on the transmission of high-risk alleles within pedigrees [19]–[23]), and to alternative previously-proposed approaches based on extending standard case/control tests (such as the Armitage trend test) to allow for either known [24], [25] or known and unknown [26] relatedness. The programs compared (see Table 1) differ in the precise details of the methodology implemented (such as whether an LMM approach is used, and, if so, whether an exact method or an approximation is used) and through various user-chosen options such as the specific method and number of SNPs used to estimate the kinship matrix. We investigate the sensitivity to these choices and the success (or otherwise) of the approaches in controlling the overall genome-wide error-rate in both real and simulated data (into which artificial simulated disease loci have been inserted).

**Table 1:** Summary of methods/software packages investigated.

Package/method and version	Approach	Kinship estimation method	Reference(s)
EMMAX emmax-intel-binary-20120210.tar.gz	LMM (approximate)	Kinship matrix estimated internally using user-supplied set of SNPs, or set to theoretical/estimated values calculated externally	[1]
FaST-LMM v2.04	LMM (approximate or exact)	Kinship matrix estimated internally using user- supplied set of SNPs, using SNPs selected through FaST-LMM-Select procedure, or set to theoretical/estimated values calculated externally	[4] [30] [31]
GEMMA v0.91	LMM (exact)	Kinship matrix estimated internally using user-supplied set of SNPs, or set to theoretical/estimated values calculated externally	[16]
GenABEL v1.7-6 (FASTA)	LMM (approximate)	Kinship matrix estimated internally using user-supplied set of SNPs, or set to theoretical/estimated values calculated externally	[9] [39]
GenABEL v1.7-6 (Grammar-Gamma)	LMM (approximate)	Kinship matrix estimated internally using user-supplied set of SNPs, or set to theoretical/estimated values calculated externally	[14] [39]
GTAM (implemented in MASTOR v0.3)	LMM (approximate)	Kinship matrix calculated externally (assumed to reflect ‘known’ (theoretical) pedigree relationships)	[8]
Mendel v13.2	LMM (approximate or exact)	Kinship matrix estimated internally using theoretical pedigree relationships, estimated within estimated pedigree clusters (using all SNPs), or fully estimated (using all SNPs)	[35]
MMM v1.01	LMM (approximate or exact)	Kinship matrix estimated internally using user-supplied set of SNPs, or set to theoretical/estimated values calculated externally	[15]
FBAT v2.0.4	Transmission of alleles within pedigrees	Method by definition uses ‘known’ (theoretical) pedigree relationships	[21] [23]
MASTOR v0.3	Retrospective quantitative trait version of MQLS	Kinship matrix calculated externally (assumed to reflect ‘known’ (theoretical) pedigree relationships)	[25]
MQLS v1.5	Adjusted version of retrospective case/control test	Kinship matrix calculated externally (assumed to reflect ‘known’ (theoretical) pedigree relationships)	[24]
ROADTRIPS v1.2 (RM test)	Adjusted version of retrospective case/control test	Kinship matrix calculated externally (assumed to reflect ‘known’ (theoretical) pedigree relationships). Further correction based on genome-wide set of SNPs applied internally.	[26]

The approaches are compared via application to real and simulated data derived from a genome-wide association study of visceral leishmaniasis (VL) in 348 Brazilian families comprising 3636 individuals (1970 with both genotype and phenotype data). This Brazilian family data set was used (together with a larger Indian case/control data set) by Fakiola et al. [13] to identify, at genome-wide levels of significance, a replicable association between variants in the HLA region on chromosome 6 and visceral leishmaniasis. Although in [13] the HLA locus (analysed using the LMM package MMM [15]) did not achieve genome-wide levels of significance in the Brazilian data set alone (p-value 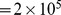), this locus was the only one to show strong evidence of association in both Brazilian and Indian data sets, and achieved convincing replication in a separate Indian cohort.

## Results

### Estimation of kinship coefficients using genome-wide SNP data

Before embarking on a detailed comparison of different methods, we explored the use of different SNP sets (containing different numbers of SNPs) for estimating pairwise kinship measures, in order to identify a robust set of SNPs that could be used for subsequent comparisons. We considered using either the full genome-wide set of SNPs (545,433 SNPs), a ‘pruned’ set of 50,129 SNPs selected to have minor allele frequencies 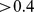 and chosen to be in approximate linkage equilibrium via the --indep 50 5 2 command in PLINK [27]), or a ‘thinned’ set of 1900 evenly-spaced SNPs that were selected from the ‘pruned’ SNPs based purely on physical position using the software package MapThin (http://www.staff.ncl.ac.uk/richard.howey/mapthin/). In addition to exploring the kinship estimates provided by various LMM software packages, we also investigated those provided by the software packages PLINK [27] and KING [28]. KING implements two different kinship estimation methods: KING-homo (KING_H), which assumes population homogeneity, and KING-robust (KING_R), which provides robust relationship inference in the presence of population substructure.

**Figure 1:**
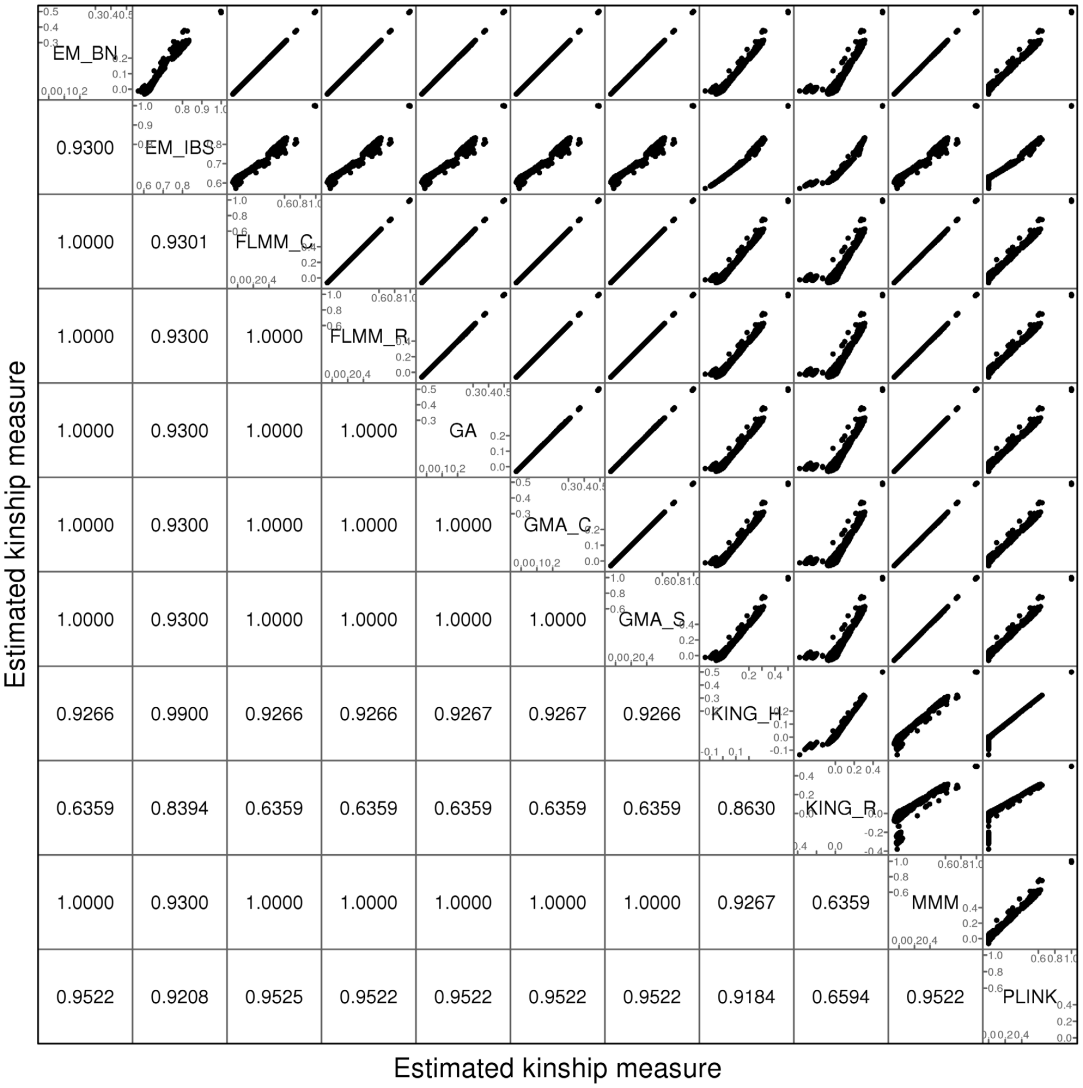
Comparison of kinship estimates (pruned SNPs) using different software packages. Plots above the diagonal show a comparison of kinship measures, with correlations between the kinship measures indicated below the diagonal. EM_BN = EMMAX (Balding-Nichols), EM_IBS = EMMAX (IBS method), FLMM_C = FaST-LMM using covariance matrix, FLMM_R = FaST-LMM using realised relationship matrix, GA = GenABEL, GMA_C = GEMMA using centred genotypes, GMA_S = GEMMA using standardised genotypes, KING_H = KING with homogeneous population assumption, KING_R = KING with robust estimation.

A comparison of the kinship estimates output by different software packages based on the pruned set of SNPs is shown in Figure 1 (similar results were seen for the full and thinned SNP sets, data not shown). Although the scale on which the kinship estimates are measured differs between different packages, the measures themselves are highly correlated, particularly those from EMMAX-BN, FaST-LMM, GenABEL, GEMMA and MMM. Kinship measures from EMMAX-IBS and PLINK were also quite well correlated, although they tended to differ slightly from those in the previous group. Kinship measures are used within the LMM framework to structure the variance/covariance matrix of the genetic random effect (see Methods). Thus, the scale of measurement (i.e. whether the kinship measure actually reflects an estimate of the kinship per se, or a rescaled measure such as twice the kinship) should not be too important, as any rescaling will be compensated for by a similar rescaling of the estimated genetic variance parameter 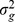 (see Methods). Kinship estimates from both KING methods tended to differ most from the other methods, with the frequent output of negative kinship estimates (compared to most other methods for which the kinship estimates are bounded at 0) among the less related individuals. This was more pronounced for KING_R than for KING_H. We consider later the possible implications of these (rather small) differences in estimated kinships for subsequent association testing.

**Figure 2:**
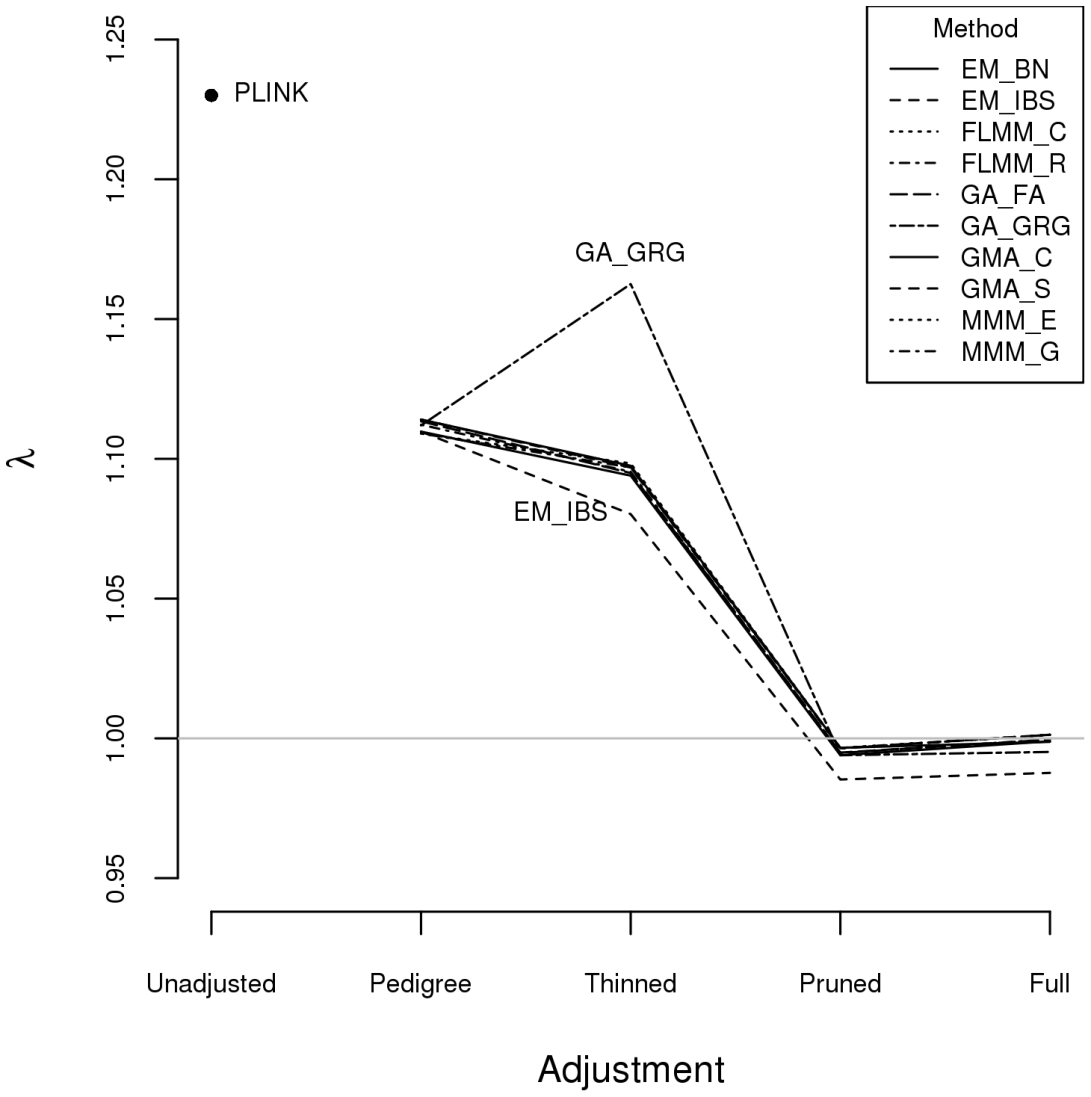
Genomic control factors obtained using different software packages and different strategies for modelling kinships. PLINK =  analysis in PLINK with no adjustment made for relatedness. Other methods/software packages are listed in Table 1 (see Table 2 for abbreviated names of methods). Pedigree  =  theoretical kinships based on known pedigree relationships used to adjust for relatedness. Thinned  =  kinships based on 1900 ‘thinned’ SNPs used to adjust for relatedness. Pruned  =  kinships based on 50,129 ‘pruned’ SNPs used to adjust for relatedness. Full  =  kinships based on 545,433 SNPs used to adjust for relatedness.

Within any given method, we found the kinship measures (for each pair of individuals) and p-values obtained (in the real data set) based on the full SNP set to be very similar to those based on the pruned set, whereas those calculated based on the thinned set were less similar (see Figure S1). The performance of the different SNP sets in terms of controlling the genome-wide type 1 error rate (i.e. controlling the genomic inflation factor 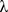 [29] to the desired level of 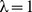) in the real data set is shown in Figure 2 (see Figure S2 for full QQ plots). All packages performed well when using the full or pruned set of SNPs (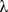 = 0.99–1.00), but performance deteriorated when the thinned set was used (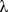 mostly about 1.08–1.10). This was most pronounced for GenABEL (GRAMMAR-Gamma), for which 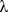 was 1.16. Our intuition is that, although 1900 SNPs may be sufficient to accurately model close relationships (such as full sib or parent-offspring), many more SNPs will be required to accurately model distant relationships within pedigrees (such as cousins, second cousins, third cousins etc.) or even more distant relationships between pedigrees. Results obtained using theoretical kinships were inflated for all methods (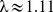), suggesting the presence of additional relatedness/population structure that is not well accounted for by known family relationships. Regardless of the method or SNP set used, adjustment always resulted in substantially lower inflation than was seen (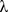 = 1.23) in unadjusted analysis.

Listgarten et al. [30] proposed an automated method, FaST-LMM-Select, to select the most appropriate set of SNPs to use for kinship estimation when testing for association in a LMM framework. The method proceeds by ordering SNPs according to their linear regression p-values and then constructing kinship matrices with an increasing number of ordered SNPs, until the first minimum genomic control factor 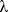 is obtained. We investigated this strategy within the FaST-LMM package using either the full or pruned set of SNPs as a starting point (see Figure S3). We found that the first minimum genomic control factor (achieved using 3–10 ordered SNPs) was generally higher than the desired value of 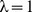, the genomic control factor subsequently decreased to considerably less than 1, and then increased back to 1 once all (pruned or full) SNPs had been included.

The automated version of FaST-LMM-Select available as an option within the current version of the FaST-LMM package uses a slightly different strategy involving *k*-fold cross-validation [31], with the ordering of SNPs and calculation of genomic control factors as varying numbers of SNPs are included in the kinship calculation carried out within the training data (and then used to predict the test data) within each cross-validation fold. The final number of SNPs to be used in the kinship calculation for the entire data set is that which minimizes the mean-squared error summed over all folds. (See FaST-LMM documentation and [31] for more details). Lippert et al. [31] found this procedure to show some advantage over using all SNPs (including a large number of presumably irrelevant SNPs) in simulations that included population stratification (but not familial relatedness) of quantitative phenotypes in randomly ascertained individuals. Application of this automated procedure to the real disease phenotype in our highly ascertained set of Brazilian pedigrees resulted in no SNPs selected for calculation of kinships when applied to the full SNP set, or two SNPs selected when applied to the pruned SNP set, resulting in a genomic control value of 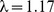 when these two SNPs were used to adjust for relatedness in the subsequent association analysis. We conclude that, at least for our data set, there is no particular advantage in using the FaST-LMM-Select procedure, indeed this procedure seems to work less well than simply using all pruned or full SNPs for estimating pairwise kinships. For the remainder of the manuscript we therefore focus on results obtained using the pruned set of SNPs to estimate kinships (apart for genome-wide analysis in the program Mendel, which by default always uses the entire set of SNPs that has been read in).

### Comparison of LMM and alternative analysis approaches

We compared the performance of the different LMM and alternative approaches listed in Table 1 through their application to real and simulated data derived from the Brazilian family data set of Fakiola et al. [13]. The simulation scenarios (see Methods) included a binary disease trait influenced by either two strong (sim-D1) or two weak (sim-D2) genetic effects or a quantitative trait (sim-Q) influenced by two strong genetic effects. In all cases the genetic effects were governed by two SNPs (rs9271252 and rs233722) located on chromosomes 6 and 12 respectively. In addition to the effects at rs9271252 and rs233722, we also allowed for 22 weaker ‘polygenic’ effects caused by genotype at the 100th SNP on each autosomal chromosome. Where applicable, we used either the default analysis options within each program, or else explored the use of different options as indicated below. The program FaST-LMM uses either maximum likelihood (ML) or restricted maximum likelihood (REML). (In early versions of FaST-LMM the default was ML but in later versions the default became REML). After some experimentation, we deemed the ML option to be the most reliable in the presence of strong genetic effects, and have therefore used ML for all results presented here.

The success of the various approaches in controlling the overall genome-wide type 1 error rate (i.e. controlling the genomic inflation factor [29] 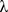 to the desired level of 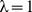) is shown in Table 2. All methods that made use of estimated kinships performed well, apart from Mendel when estimation was restricted only to estimated pedigree clusters (which gave 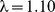) and MQLS, for which use of estimated kinships (in the 1972 genotyped individuals) appeared to result in slightly deflated genomic inflation factors. For all other methods, use of estimated kinships reduced the genomic inflation factor to around 1, compared to a value of 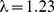 in the real data (and up to 1.43 in the simulated data) when performing an unadjusted analysis. Methods that used only theoretical kinships based on ‘known’ pedigree information performed well in the simulated data sets, but were less successful at controlling inflation for the real data set, suggesting that our real data contains additional, more complicated, relatedness or population substructure that is not accounted for by known family relationships.

**Table 2:** Genomic control inflation factors achieved in real data or in a single replicate of the simulated data sets.

			Trait analysed			
Method	Description	Kinships used	Real disease (VL)	Simulated strong (sim-D1)	Simulated weak (sim-D2)	Simulated quantitative (sim-Q)
Unadjusted	Standard linear or logistic regression	None	1.23	1.12	1.04	1.43
EM_BN	EMMAX (Balding-Nichols kinships)	Estimated	0.99	0.99	1.00	0.99
EM_IBS	EMMAX (IBS kinships)	Estimated	0.99	0.99	1.00	1.00
FLMM_A	FaST-LMM (approximate calculation)	Estimated	0.99	0.99	1.00	1.00
FLMM_E	FaST-LMM (exact calculation)	Estimated	1.00	0.99	1.01	1.00
GA_FA	GenABEL (FASTA)	Estimated	0.99	0.99	1.00	0.99
GA_GRG	GenABEL (GRAMMAR-Gamma)	Estimated	0.99	0.99	1.00	1.00
GMA_C	GEMMA using centred genotypes	Estimated	1.00	0.99	1.01	1.00
GMA_S	GEMMA using standardised genotypes	Estimated	1.00	0.99	1.01	1.00
GTAM	GTAM (implemented in MASTOR)	Pedigree	1.20	1.00	0.99	0.99
Mendel_T	Mendel with theoretical kinships	Pedigree	1.11	1.00	0.99	0.99
Mendel_P	Mendel with kinships estimated within estimated pedigree clusters	Estimated	1.10	1.00	0.99	0.99
Mendel	Mendel with fully estimated kinships	Estimated	1.03	0.99	1.00	1.00
MMM_E	MMM (exact calculation)	Estimated	1.00	0.99	1.01	1.00
MMM_G	MMM (GLS approximation)	Estimated	0.99	0.99	1.00	0.99
FBATaff^a^	FBAT (transmissions to affecteds only)	Pedigree	1.02	1.01	1.00	–
FBATboth	FBAT (transmissions to all individuals)	Pedigree	1.01	1.00	1.01	1.00
MASTOR	MASTOR (implemented in MASTOR)	Pedigree	1.15	1.00	0.99	0.99
MQLS1972^a^	MQLS (using 1972 genotyped individuals)	Pedigree	1.15	1.01	0.99	–
MQLS3626^a^^,^ b	a ^,^ bMQLS (using all 3626 individuals with or without genotype data)	Pedigree	1.16	–	–	–
MQLS1972_E	MQLS using 1972 genotyped individuals and estimated kinships	Estimated	0.94	0.90	0.91	–
RT1972^a^	ROADTRIPS (using 1972 genotyped individuals)	Pedigree & estimated	1.00	1.00	0.99	–
RT3626^a^^,^ b	ROADTRIPS (using all 3626 individuals with or without genotype data)	Pedigree & estimated	1.00	–	–	–

a FBATaff, MQLS and ROADTRIPS are only applicable to binary traits and so do not have results in the ‘Simulated quantitative’ column.

b In the simulated data sets, MQLS and RT could only be based on the 1972 individuals with simulated phenotypes, and so no simulated trait results are displayed in the MQLS3626 and RT3626 rows.

The Brazilian populations studied here are believed to be long-term (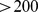 years) admixtures of Caucasian, Negroid and Native Indian ethnic backgrounds, as confirmed in recent analysis of a subset of our families [32]. The discrepancy between the genomic inflation factors seen in our real and simulated data results suggests that our (relatively simplistic) simulation scenarios have not been able to fully mimic the underlying population structure existant in the real data; although our simulation strategy (see Methods) was designed to generate trait correlations that reflect close familial relationships, we did not specifically endeavour to generate correlations due to population stratification or more distant/cryptic relationships. To investigate the relative contributions of phenomena such as admixture/population stratification/cryptic relationships to the inflation observed in our real data when using theoretical (pedigree-based) kinships, we applied the ADMIXTURE program [33] to our pruned set of SNPs to estimate ancestry proportions (assuming 3 ancestral populations) in each individual. Although the variation in ancestry proportion estimated within each individual was quite large (standard deviation 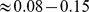 depending on ancestral population) there was no evidence (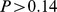) for a relationship between estimated ancestry proportion and disease status, suggesting that the inflation in test statistics observed when using theoretical kinships is more likely to be due to unmeasured cryptic relationships and/or subtle population substructure, than to population substructure or admixture directly related to the Caucasian, Negroid and Native Indian ethnicities. This conclusion was supported by the fact that logistic regression analysis allowing for the ancestry proportions as covariates resulted in a genomic control inflation factor of 1.17, only slightly reduced from the unadjusted genomic control inflation factor of 1.23.

We also used as covariates in a logistic regression analysis the first nine coordinates obtained from a multidimensional scaling (MDS) analysis of the pruned SNPs in PLINK (having considered between one and ten coordinates, nine was the number that minimised the genomic control inflation factor). The resulting genomic control inflation factor was 1.08, considerably smaller than the unadjusted inflation factor of 1.23, but still not perfectly controlled. Inclusion of MDS coordinates as covariates, similar to including principal components scores, might be expected to account for more subtle levels of population substructure than are accounted for by the use of the ADMIXTURE program (and may possibly also indirectly account for relatedness), which perhaps explains the greater success of this procedure. However the fact that LMM approaches based on estimated kinships still do better (with respect to controlling 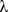) than does the MDS approach suggests there may still be levels of known or cryptic relatedness that are not well-captured by these first nine coordinates.

**Figure 3:**
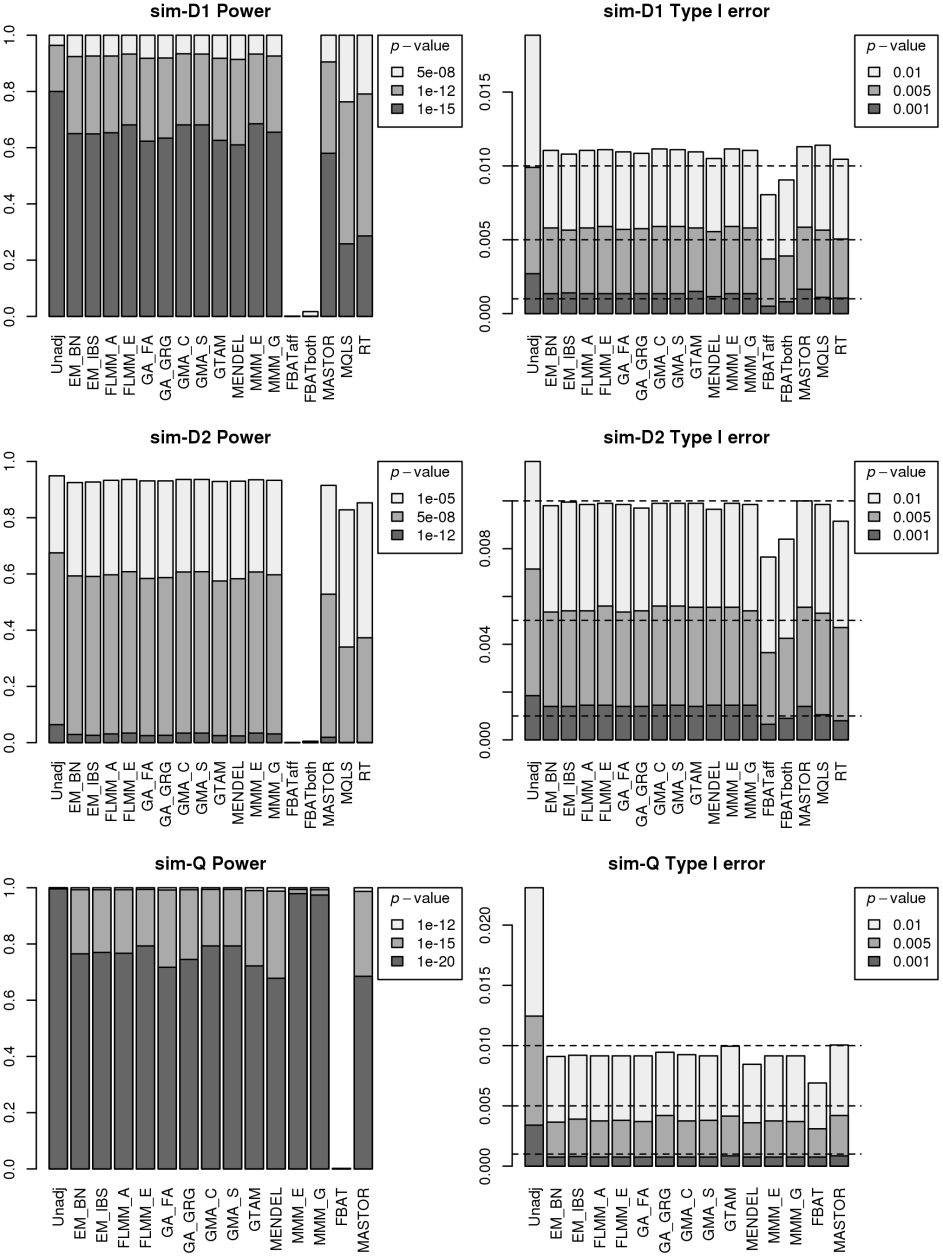
Power and type 1 error of different methods. Powers (left hand plots) are defined as the proportion of replicates (out of 1000) in which both simulated disease loci are detected, with ‘detection’ corresponding to any SNP within 40 kb of the simulated disease locus reaching the specified *p*-value threshold. Type 1 errors (right hand plots) are defined as the proportion of null SNPs (out of 20,000 = 20 null SNPs times 1000 simulation replicates) that reach the specified *p*-value threshold. Horizontal dashed lines indicate the target *p*-value thresholds (i.e. the expected type 1 error rates).

**Figure 4:**
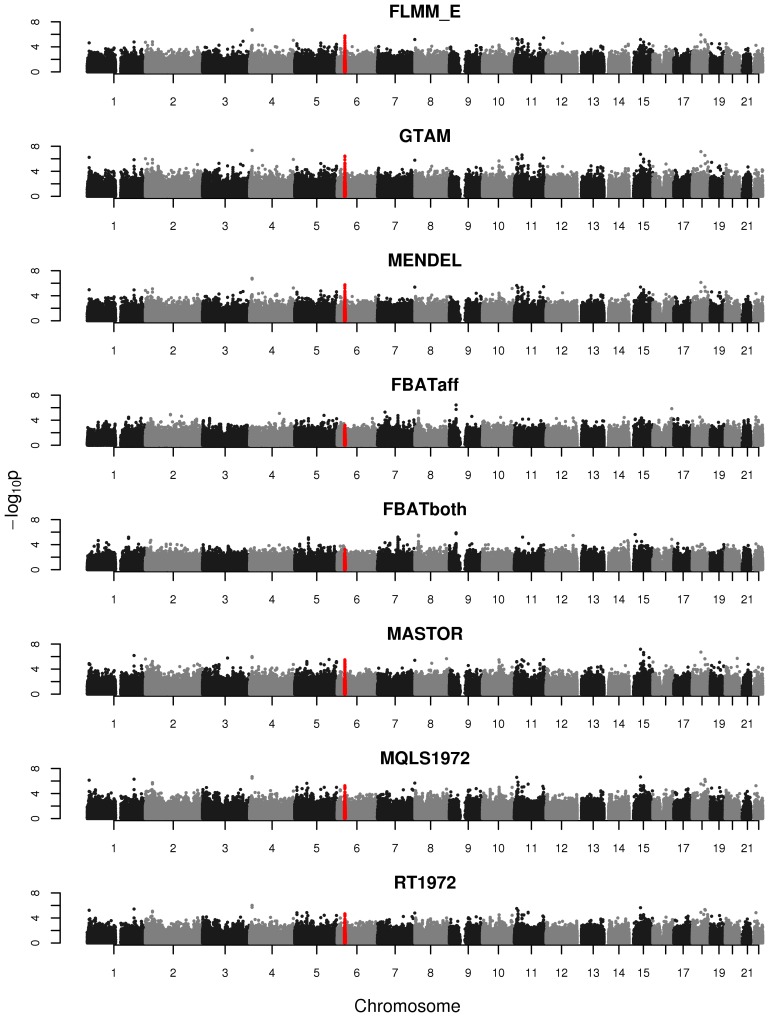
Manhattan plots for the real phenotype using FaST-LMM exact and alternative software packages. The points marked in red denote the confirmed significant region from Fakiola et al. (2013). FLMM_E = FaST-LMM using exact calculation, MQLS1972 = MQLS using 1972 genotyped individuals, RT1972 = ROADTRIPS using 1972 genotyped individuals, FBATaff = FBAT using transmissions to affecteds only, FBATboth = FBAT using transmissions to both affecteds and unaffecteds. Results from all other LMM methods were indistinguishable from FLMM_E and so are not shown.

An intuitive overview of the expected power provided by the different (real and simulated) data sets can be obtained from Figure S4, which shows Manhattan plots from a FaST-LMM analysis of a single replicate of real or simulated data. The real phenotype data shows a noticeable signal in the HLA region on chromosome 6, consistent with the main finding in [13], while for all simulated traits the primary associated regions are correctly identified without any obvious false signals. A formal comparison of power and type 1 error for the different analysis methods using 1000 simulation replicates is shown in Figure 3. All methods apart from an unadjusted analysis show acceptable levels of type 1 error (although note that the type 1 error rate for FBAT appears to be slightly conservative). In terms of power, all LMM approaches (including GTAM and Mendel) and MASTOR show similar performance, apart from MMM which shows slightly higher power than other methods for detection of loci involved in the (strong) simulated quantitative trait. ROADTRIPS and MQLS show slightly lower power than the LMM approaches, while the approaches implemented in FBAT appear to be considerably less powerful than those implemented in the LMM and other packages (even allowing for FBAT's slightly conservative levels of type 1 error). The lower power of FBAT is likely to be caused by the smaller effective sample size (357 cases compared to 357 ‘pseudo’ controls in FBAT, versus 357 cases compared to 1613 genuine controls in the LMM and other alternative approaches), due to the way the FBAT test statistics are constructed. These results are consistent with a visual examination of the Manhattan plots obtained from the different methods using either the real data or a single replicate of the simulated data (Figure 4, Supplementary Figures S5–S6), with FBAT achieving much lower levels of significance around the true or simulated phenotype-associated SNPs than do the other methods. (The results from all LMM methods not displayed in Figure 4 and Supplementary Figures S5–S6 were indistinguishable from FLMM_E, data not shown).

Although the LMM (and several alternative) approaches show similar overall levels of power, an interesting separate question is the degree of concordance between the different methods with respect to the association signals detected. In the real data set we found the p-values obtained at each SNP from the different LMM methods to be highly concordant (Figure S7), while the concordance between the LMM methods and alternative approaches (Figure S8) is high for all methods other than FBAT (although lower than is observed among methods within the LMM class). The test implemented in FBAT is statistically uncorrelated with that implemented in the LMM and other alternative approaches, therefore it is not surprising that little concordance is seen between the test statistics achieved at the vast majority of (presumably null) SNPs. Figure S8 also shows that methods that use phenotype information from non-genotyped family members (MQLS3626 and RT3626, which use all 3626 individuals regardless of whether or not they have genotype data) are most similar to each other and less similar to methods that use information only from the genotyped individuals.

The high concordance between the different LMM methods (and, to a slightly lesser extent, between LMM methods and all methods other than FBAT) is also seen for the simulated (weak disease) trait (Figure S9); similar results were found for the other simulated traits and other LMM methods (data not shown). A formal comparison of the concordance between ‘top hits’ identified by the different methods in the simulated data (1000 simulation replicates, comparison restricted to true and null simulated regions) is shown in Table 3. Using EM_BN as reference, the concordance between the top SNPs identified is seen to be extremely high for all other methods except FBAT, suggesting again that all methods except FBAT provide essentially the same inference.

**Table 3:** Concordance between top SNPs identified by different methods.

		Mean (standard deviation) in 1000 replicates of proportion of top *t* SNPs within null and true regions that overlap with top *t* SNPs from EM_BN				
Trait	Method^a^	*t* = 5	*t* = 10	*t* = 15	*t* = 20	*t* = 25
sim-D1	Unadjusted	0.991 (0.042)	0.990 (0.030)	0.981 (0.033)	0.975 (0.032)	0.973 (0.027)
	EM_IBS	0.999 (0.017)	0.999 (0.009)	0.997 (0.015)	0.997 (0.013)	0.996 (0.012)
	FLMM_A	1.000 (0.009)	1.000 (0.003)	1.000 (0.007)	1.000 (0.004)	1.000 (0.003)
	FLMM_E	0.998 (0.021)	1.000 (0.005)	0.999 (0.008)	0.999 (0.005)	1.000 (0.004)
	GA_FA	0.998 (0.018)	1.000 (0.005)	0.999 (0.011)	0.999 (0.008)	0.998 (0.008)
	GA_GRG	0.998 (0.021)	0.999 (0.011)	0.996 (0.017)	0.998 (0.010)	0.998 (0.008)
	GMA_C	0.998 (0.021)	1.000 (0.004)	0.999 (0.009)	0.999 (0.005)	1.000 (0.004)
	GMA_S	0.998 (0.021)	1.000 (0.005)	0.999 (0.008)	0.999 (0.005)	1.000 (0.004)
	GTAM	0.998 (0.022)	0.995 (0.022)	0.990 (0.025)	0.988 (0.022)	0.987 (0.020)
	Mendel	0.997 (0.025)	0.996 (0.019)	0.991 (0.024)	0.989 (0.021)	0.989 (0.018)
	MMM_E	0.991 (0.041)	1.000 (0.004)	0.999 (0.009)	0.999 (0.005)	1.000 (0.004)
	MMM_G	0.993 (0.036)	1.000 (0.003)	1.000 (0.007)	1.000 (0.005)	0.999 (0.005)
	FBATaff	0.684 (0.253)	0.790 (0.115)	0.773 (0.090)	0.771 (0.080)	0.760 (0.072)
	FBATboth	0.859 (0.130)	0.844 (0.084)	0.811 (0.078)	0.795 (0.075)	0.777 (0.071)
	MASTOR	0.993 (0.038)	0.994 (0.024)	0.989 (0.027)	0.985 (0.024)	0.985 (0.022)
	MQLS	0.978 (0.062)	0.981 (0.040)	0.960 (0.043)	0.951 (0.041)	0.941 (0.038)
	RT	0.981 (0.059)	0.984 (0.037)	0.962 (0.042)	0.952 (0.041)	0.942 (0.038)
sim-D2	Unadjusted	0.982 (0.060)	0.984 (0.041)	0.979 (0.039)	0.974 (0.040)	0.973 (0.036)
	EM_IBS	0.997 (0.029)	0.997 (0.024)	0.995 (0.025)	0.994 (0.028)	0.994 (0.024)
	FLMM_A	0.998 (0.027)	0.998 (0.024)	0.997 (0.025)	0.997 (0.029)	0.997 (0.026)
	FLMM_E	0.995 (0.035)	0.997 (0.025)	0.997 (0.025)	0.996 (0.030)	0.997 (0.026)
	GA_FA	0.992 (0.044)	0.998 (0.024)	0.997 (0.026)	0.996 (0.030)	0.996 (0.026)
	GA_GRG	0.994 (0.038)	0.997 (0.026)	0.996 (0.027)	0.995 (0.030)	0.996 (0.026)
	GMA_C	0.995 (0.035)	0.997 (0.025)	0.997 (0.025)	0.996 (0.030)	0.997 (0.026)
	GMA_S	0.995 (0.035)	0.997 (0.025)	0.997 (0.025)	0.996 (0.030)	0.997 (0.026)
	GTAM	0.988 (0.050)	0.990 (0.036)	0.983 (0.037)	0.982 (0.036)	0.982 (0.032)
	Mendel	0.988 (0.051)	0.992 (0.033)	0.986 (0.035)	0.984 (0.036)	0.987 (0.031)
	MMM_E	0.995 (0.037)	0.997 (0.025)	0.997 (0.025)	0.996 (0.030)	0.997 (0.026)
	MMM_G	0.998 (0.028)	0.998 (0.024)	0.997 (0.025)	0.997 (0.029)	0.997 (0.026)
	FBATaff	0.413 (0.255)	0.571 (0.201)	0.614 (0.157)	0.639 (0.128)	0.651 (0.102)
	FBATboth	0.664 (0.246)	0.718 (0.146)	0.699 (0.111)	0.691 (0.099)	0.686 (0.088)
	MASTOR	0.971 (0.075)	0.988 (0.038)	0.981 (0.038)	0.978 (0.039)	0.979 (0.033)
	MQLS	0.934 (0.107)	0.962 (0.056)	0.942 (0.053)	0.928 (0.051)	0.917 (0.047)
	RT	0.943 (0.099)	0.965 (0.055)	0.943 (0.053)	0.930 (0.052)	0.919 (0.047)
sim-Q	Unadjusted	0.987 (0.049)	0.983 (0.038)	0.962 (0.040)	0.963 (0.034)	0.954 (0.033)
	EM_IBS	0.998 (0.020)	0.998 (0.016)	0.993 (0.020)	0.994 (0.017)	0.993 (0.015)
	FLMM_A	1.000 (0.000)	1.000 (0.000)	1.000 (0.004)	1.000 (0.005)	1.000 (0.004)
	FLMM_E	1.000 (0.009)	0.999 (0.008)	1.000 (0.005)	1.000 (0.005)	0.999 (0.005)
	GA_FA	1.000 (0.006)	0.999 (0.010)	0.998 (0.010)	0.998 (0.010)	0.996 (0.012)
	GA_GRG	0.994 (0.034)	0.999 (0.010)	0.995 (0.018)	0.996 (0.014)	0.996 (0.012)
	GMA_C	1.000 (0.009)	1.000 (0.007)	1.000 (0.004)	1.000 (0.004)	1.000 (0.004)
	GMA_S	1.000 (0.009)	0.999 (0.008)	1.000 (0.005)	1.000 (0.005)	0.999 (0.005)
	GTAM	0.995 (0.032)	0.991 (0.028)	0.984 (0.030)	0.985 (0.024)	0.984 (0.022)
	Mendel	0.998 (0.021)	0.996 (0.020)	0.987 (0.027)	0.988 (0.022)	0.988 (0.019)
	MMM_E	0.899 (0.100)	0.999 (0.008)	1.000 (0.004)	1.000 (0.004)	1.000 (0.004)
	MMM_G	0.903 (0.100)	1.000 (0.003)	1.000 (0.003)	1.000 (0.004)	1.000 (0.003)
	FBAT	0.906 (0.101)	0.896 (0.067)	0.869 (0.059)	0.844 (0.067)	0.814 (0.066)
	MASTOR	0.998 (0.020)	0.992 (0.027)	0.984 (0.030)	0.984 (0.025)	0.983 (0.023)

a See Table 2 for description of methods.

### Feeding externally estimated kinship coefficients into LMMs

Most LMM packages (although not Mendel) allow a separation between the ‘estimation of kinships’ step and the ‘association testing’ step. This is convenient as it allows the user to read in theoretical or estimated kinships as desired, and to consider using an alternative package for estimating kinships to the one used for the actual association testing. We investigated performing an analysis in FaST-LMM (exact calculation), but with the kinships estimated from various different software packages (see Figure S10 and Table S1). Use of the ‘wrong’ kinship estimates (chosen to be inversely related to the theoretical kinship value) resulted in very similar results to unadjusted analyses (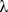 = 1.23 in the real trait, 1.12 in the simulated strong disease trait, and 1.43 in the simulated quantitative trait). Results based on kinship estimates from KING_R and KING_H were very similar to those obtained using FaST-LMM's own realised relationship matrix (FLMM-R) for all traits, and provided good control of the genome-wide error rate (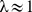) in spite of the unusual pattern in KING's estimated kinships that had been noted in Figure 1. Estimation of kinships using PLINK was less satisfactory, leading to inflated genomic control factors in both real and simulated data sets. This is consistent with previous results [28] suggesting that PLINK performs less well than KING for relationship estimation. Interestingly, although KING_R has been shown to have an advantage over KING_H in non-homogeneous populations when the goal is relationship estimation for its own sake [28], this advantage is not apparent here, where the goal is instead to adjust for potentially different levels of relatedness, from close family relationships to more distant relationships (perhaps mimicking population membership), while performing association testing.

### Computational efficiency and ease-of-use

Given that many of the software implementations we investigated (and in particular all the various LMM implementations) showed similar levels of power and type 1 error, and gave rather similar inference in terms of localisation of signals and 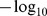 p-values achieved, an important practical consideration when deciding what implementation to use is the ease-of-use and computational efficiency. Ease-of-use is necessarily somewhat subjective as it depends on a user's prior experience and software/operating system preferences. Computational efficiency can, in theory, be examined more objectively, however, in practice, the total time required to perform an analysis is dependent on the computer architecture available (in particular the ability of the system and of any given program to allow multi-threading), demands of competing users and the availability of (and ability of any given program to make use of) facilities for parallel processing e.g. a multi-node compute cluster. These considerations make it hard to perform a genuine ‘head-to-head’ comparison between different packages. In Table S2 we present an approximate comparison (carried out on the same machine, without use of parallel processing) together with some comments concerning ease-of-use. Since many groups (including ourselves) use PLINK [27] to perform initial quality control of genome-wide association data, we considered programs that could use PLINK files directly (or with just a few easily-implemented transformation steps) to be the easiest to use, while those programs that required more extensive data transformation, creation of additional input files and/or external estimation of kinships were considered harder.

With respect to computational speed, as a rule of thumb we found Mendel (theoretical kinships), FaST-LMM (approximate) and GenABEL (GRAMMAR-Gamma) to be the fastest LMM implementations, taking between 3 minutes and a quarter of an hour on our system to analyse 545,433 SNPs in 1972 genotyped individuals. These were closely followed by EMMAX and MMM (approximate) which took around half an hour, GenABEL (FASTA), GEMMA, FaST-LMM (exact) and MMM (exact) which typically took 1–2 hours, Mendel (estimated kinships) which took around 2.5 hours, and GTAM which took around 4 hours. Of the non-LMM methods, FBAT, MQLS and MASTOR were the fastest, taking a few hours to perform the analysis, while ROADTRIPS was the slowest, taking several days. Inputting estimated (rather than theoretical) kinships into MQLS increased the time taken to around 4 days (and appeared to over-correct the genomic inflation, see Table 2), while an analysis inputting estimated (rather than theoretical) kinships into ROADTRIPS was still running (with analysis completed for only 38,926 of the desired 545,433 SNPs) after more than 2 months. Neither MQLS nor ROADTRIPS were designed for analysis of unrelated individuals and so are most likely optimised for reading in and working with relatively sparse kinship matrices (in which individuals from different pedigrees are assumed to have kinships equal to 0); to force the programs to consider estimated kinships between all individuals we had to recode the pedigree names to pretend that everyone comes from the same pedigree, which most likely considerably increases processing and memory requirements.

### Analysis of longitudinal phenotypes

Eu-ahsunthornwattana et al. [18] investigated a strategy for analysing longitudinal traits (repeated measures) in a linear mixed model framework simply by treating each measurement as if it came from a different individual, and expanding out the genetic data set accordingly (resulting in an expanded data set containing many apparent twins, triplets, quadruplets etc., depending on how many measurements are available for each person). We investigated this strategy in the current data set using a single replicate of data (498 individuals) simulated under either a longitudinal (sim-L20) or longitudinal polygenic (sim-P20) model (see Methods). Results (Table 4) showed that EMMAX, FaST-LMM and GEMMA were successful in maintaining the genomic inflation factor to about 1, whereas GenABEL (FASTA) and MMM showed some inflation, particularly in the polygenic longitudinal simulation, and GenABEL (GRAMMAR-Gamma) showed strong *deflation*. Comparison of the concordance in 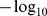 p-values achieved by the different methods (data not shown) indicated that, although the results from different methods were highly correlated (in terms of the top SNPs identified), the actual p-values achieved were very different, consistent with the differences seen in overall distribution of test statistics.

**Table 4:** Genomic control factors achieved in naive analysis of a single replicate of the simulated longitudinal data sets.

	Trait analysed	
Method^a^	Longitudinal (sim-L20)	Longitudinal polygenic (sim-P20)
Unadjusted	20.82	21.53
EM_BN	1.01	1.01
EM_IBS	0.99	0.97
FLMM_A	1.01	1.01
FLMM_E	1.01	1.01
GA_FA	1.06	2.39
GA_GRG	0.66	0.47
GMA_C	1.01	1.01
GMA_S	1.01	1.01
MMM_E	1.01	3.52
MMM_G	1.01	3.52

a See Table 2 for description of methods.

Analysing each repeated measure as if it comes from a different individual treats our data set as a larger ‘pseudo data set’ containing many apparent twins/triplets/quadruplets (actually, in this case, 20-tuplets). Although less satisfactory than a proper longitudinal analysis that takes into account correlations due to both relatedness between individuals and repeated measures within individuals [34], our intuition was that the LMM framework would absorb the effect of repeated measures within individuals into the genetic component of variance estimated, resulting in an overall correct distribution of test statistics. For EMMAX, FaST-LMM and GEMMA, this intuition appears to have been correct. Although for GenABEL (FASTA) and MMM the resulting distribution of test statistics is inflated, the linear relationship between the observed and desired test statistics means that test statistics following the desired distribution could be obtained simply by dividing the observed 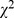 test statistics by the observed genomic control inflation factor, in an approach akin to standard genomic control [29].

We also investigated a ‘proper’ longitudinal analysis implemented within the R software package longGWAS [34]. QQ plots from longGWAS (data not shown) indicated acceptable genomic control inflation factors (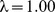 and 0.97 for sim-L20 and sim-P20 respectively). A comparison of longGWAS with our (improper) approach using FaST-LMM (data not shown) indicated that the results (in terms of the 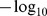 p-values obtained at each SNP) from longGWAS and FaST-LMM were highly correlated for both sim-L20 and sim-P20. Although the ‘proper’ analysis implemented in longGWAS might be considered theoretically most appealing, we note that longGWAS was considerably slower than FaST-LMM, taking approximately 19 hours (in comparison to 5.5 minutes for FaST-LMM), when run in parallel for each of 22 chromosomes. If run as a single process (all chromosomes), this translates to about 9.5 days for longGWAS versus 7.6 hours for FaST-LMM. Thus, given the satisfactory performance of FaST-LMM, and the high correlation between the results obtained from FaST-LMM and those from longGWAS, from a practical point of view, FaST-LMM (or possibly EMMAX or GEMMA) would seem the more attractive option.

Another program that can, in theory, implement a ‘proper’ longitudinal analysis is the lmekin function within the R package coxme. We found this function to be computationally infeasible for analysis of genome-wide data, but application to a selected set of 2423 SNPs (of different effect sizes) in the sim-L20 data suggested that the results were very similar to those obtained from GenABEL (FASTA), EMMAX, FaST-LMM, GEMMA and MMM. However, we were unable to get lmekin to give meaningful results (most results were “NA”) when applied to the sim-P20 data. We also speculated that a ‘proper’ longitudinal analysis should, in theory, be implementable in the package Mendel [35], through making use of Mendel's ability to include household effects. (Effectively one would trick Mendel into fitting the correct model by designating all ‘individuals’ (with each timepoint considered as a separate individual) to be members of a single pedigree, with the individuals corresponding to separate timepoints within a single real individual designated as belonging to the same household). We attempted to fit this model in Mendel for our sim-L20 and sim-P20 data sets, but were unable to obtain reliable results. (If included, household effects were continually estimated at 0, and, regardless of whether or not household effects were included, the SNP association tests showed highly inflated significance values, with no correct localisation of true sim-L20 signals as had been seen for FaST-LMM (Figure S4) and little correlation between 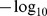 p-values from Mendel and those from these other packages). We speculate that the algorithm used by Mendel may be adversely affected by the presence of many highly-related individuals (e.g. repeated measures that in actuality pertain to a single individual), causing the test statistics generated to be unreliable.

## Discussion

Here we have demonstrated, through simulations and application to real data, that linear mixed model approaches such as those implemented in the packages GenABEL, EMMAX, FAST-LMM, GEMMA and MMM offer a convenient and robust approach for family-based GWAS of quantitative or binary traits, are successful in controlling the overall genomic inflation factor to an appropriate level, and offer higher power than traditional family-based association analysis approaches such as those implemented in FBAT. Similar inference is also provided by related and alternative approaches implemented in the software packages Mendel, ROADTRIPS, MQLS and MASTOR, although our results from analysis of the real data suggest that, for Mendel, MQLS and MASTOR, care may need to be taken to use estimated kinships based on SNP data rather than known pedigree relationships, if one is to avoid any inflation in the test statistics.

Our current study focused mostly on family data in which genuine close relationships between many individuals exist. Nevertheless we found similar results with respect to the LMM methods investigated (adequate control of type 1 error and extremely similar performance in terms of power and concordance between top findings) when applied to a subset of 462 founder individuals from our pedigrees, selected to be approximately unrelated to one another (see Figure S11 and Table S3). Therefore, we believe that our results highlighting the concordance between different LMM methods are equally relevant to researchers carrying out genome-wide association studies of apparently unrelated individuals as to researchers carrying out family-based studies.

Traditional methods for family-based association analysis make use of pedigree relationships either (e.g. FBAT) through direct use of known pedigree structure or else (e.g. MQLS, ROADTRIPS and all LMM methods) through use of a covariance matrix that involves the known kinship between each pair of individuals (the probability that a randomly chosen allele at a locus in each individual is identical by descent i.e. is a copy of a common ancestral allele, under the assumption that the pedigrees are correctly specified and all founders in a pedigree are completely unrelated i.e. share no alleles identical by descent). The assumption that all founders in a pedigree share no alleles identical by descent is clearly a fiction, given human population history, while the assumption that all pedigrees are correctly specified and unrelated to one another is also likely to be violated in most real studies. The use of estimated kinships based on SNP data rather than theoretical kinships based on known pedigree relationships removes the reliance on these untenable assumptions, and allows essentially the same analysis approaches to be applied to apparently unrelated individuals (who may nevertheless display distant levels of shared ancestry). The question then arises as to what exactly these estimated kinships (or related measures) are actually measuring? We consider a detailed discussion of this issue to be beyond the scope of the current manuscript, but we refer the reader to the more detailed expositions given in [36] and [37] which discuss some differences between different kinship measures as well as pointing out the difficulty of directly modelling identity by descent in the absence of an explicit pedigree. A key point when using estimated kinships to structure the covariance matrix in an association analysis (as here) is that our goal is not relationship estimation (close or distant) in its own right, but rather to adjust our analysis for phenotypic correlations between individuals due to genetic factors (usually assumed to be polygenic effects) that would otherwise result in inflated association test statistics. Therefore, one could argue that the extent to which the estimated kinship measures do or do not reflect genuine relationships between individuals (and how one should interpret such relationships) is largely irrelevant; the important issue is whether or not use of such kinships succeeds with respect to adequately modelling phenotypic correlations between individuals. On that note, in the analyses performed here we did not find large differences between the results obtained using different kinship measures, although use of the kinship measures output by PLINK (as well as use of completely incorrect kinship measures) did perform worse than the other kinship measures investigated.

The recent popularity of LMM approaches for the analysis of apparently unrelated individuals [1]–[4] has been partly motivated by a desire to correct for more complicated models of population structure including population stratification, rather than (or in addition to) correcting for relatedness between individuals. Population stratification can be thought of as a type of relatedness in that members of the same sub-population are effectively more closely related to one another than to individuals in other sub-populations, although it has been noted [36] that this sub-population or ‘island model’ underlying the traditional view of population stratification may be unduly simplistic. The observation that LMM approaches have sometimes worked better than traditional principal component approaches at correcting for apparent population structure [1] may reflect the fact that the inflation seen in genome-wide test statistics (in the absence of any correction) results not from population stratification under an ‘island model’ per se, but rather from more complicated population structure (involving distant ancestral relationships between individuals). A recent paper by Wang et al. [38] showed that, in the presence of cryptic relatedness between study subjects (but no population stratification), both principal component and LMM methods are valid (in the sense of generating test statistics with the desired distribution under the null hypothesis), but LMM approaches are more powerful for detecting association. In contrast, in the presence of population stratification, neither principal component nor LMM methods are strictly valid, but LMM methods seem to display better overall performance.

An interesting finding of our current study was the fact that longitudinal traits (repeated measures) could be successfully analysed in an LMM framework simply by treating each measurement as if it came from a separate person and expanding out the genetic data set accordingly (resulting in an expanded data set containing many apparent twins, triplets, quadruplets etc.). From a practical point of view this is useful as analysis of an expanded data set in standard LMM software is computationally convenient; we found a ‘proper’ analysis using software such as longGWAS [34] to be prohibitively slow when applied to our data set.

A caveat to all the results presented here is that they relate to genotypes derived from a single data set, our Brazilian family study of visceral leishmaniasis [13]. (Although the results in terms of the performance and power of different methods were comparable across both real and simulated data sets, even in the simulated data all genotypes were held fixed and only phenotypes were re-simulated). However, we have good reason to believe that the high concordance between different LMM implementations seen here (as well as their performance from when applied naively to longitudinal data) will hold more generally for genetic studies of diverse phenotypes carried out in diverse human populations. We observed essentially the same pattern of results described here when we applied a more limited set of LMM implementations to GWAS data from Genetic Analysis Workshop 18 (959 Mexican-American individuals from 20 families, with real and simulated phenotypes) [18] as well as when we applied these approaches to GWAS data from 402 Aboriginal Australian individuals that cluster loosely into 4 large nominal pedigrees (unpublished data). Therefore, although it is possible that highly structured populations (such as those encountered in plant or animal breeding experiments) may uncover subtle differences between the various LMM approaches, for researchers carrying out complex genetic disease studies in human populations, we anticipate there will be little difference between the results seen from one approach over another, and the choice of which method/software package to use will be largely dictated by personal taste or convenience.

On this note, we point out that each package has its own particular advantages (and disadvantages). These include the fact that EMMAX, GEMMA and MMM allow the input of dosages derived from imputed (in addition to real) genotypes; MMM has the advantage of allowing the output of regression coefficients and standard errors for the SNP effects on the (log) odds ratio scale, making it convenient to compare or combine the results with results from traditional case/control studies analysed via logistic regression; GenABEL (GRAMMAR-Gamma) has the advantage of scaling linearly with sample size, which makes it attractive for the analysis of very large data sets; FaST-LMM has the advantage, along with EMMAX and Mendel, of internally imputing missing data at any (genetic or non-genetic) covariates, which can make it convenient for implementing stepwise conditional analyses; and, unlike most LMM implementations, ROADTRIPS, MQLS and MASTOR have the advantage of using all phenotype information, including that for individuals that have not been genotyped, which can in theory generate a small increase in power.

One of the main differences between the different software implementations we investigated was the time taken to perform the analysis (not including the time required to re-format data into an appropriate format for a given package). We were unable to do a strict head-to-head comparison as the precise timings depend on a number of factors including the computer architecture available (in particular the ability of the system and of any given program to allow multi-threading and/or parallel processing), however our rough comparison (Table S2), assuming that kinships are to be estimated on the basis of SNP data, implicated FaST-LMM (approximate calculation), GenABEL (GRAMMAR-Gamma) and EMMAX as generally the fastest implementations.

In conclusion, we recommend linear mixed model approaches as a convenient and powerful approach for family-based GWAS of quantitative or binary traits. We find these approaches to be successful in controlling the overall genome-wide error rate and to perform well in comparison to competing approaches.

## Materials and Methods

### Ethics statement

Ethical approval for the Belem Family Study was obtained originally from the local ethics committee at the Instituto Evandro Chagas, Belém, Para, Brazil. Approval for continued use of the Belem Family Study samples, and for collection and use of the samples from Natal, has been granted from the local Institutional Review Board at the Universidade Federal do Rio Grande do Norte (CEP-UFRN 94–2004), nationally from the Comissão Nacional de Ética em Pesquisa (CONEP: 11019), and from the Ministerios Cencia e Tecnologia for approval to ship samples out of Brazil (portaria 617; 28 September 2005). Informed written consent for sample collection was obtained from adults, and from parents of children 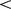
18 years old.

### Subjects and genotyping

Sample collection and genotyping of the Brazilian subjects used here is described in detail in [13]. In brief, we ascertained 348 families comprising 65 families collected from sites around Belém and 283 families collected from sites around Natal in north east Brazil. All families were ascertained on the basis of containing multiple individuals that had been diagnosed with clinical visceral leishmaniasis. DNA from 2159 family members was genotyped at the Wellcome Trust Sanger Institute using the Illumina Human660-Quad chip. Extensive quality control checks were employed to retain only high quality samples [13], and to exclude samples whose apparent relatedness (as assessed based on estimated genome-wide average identity by descent, calculated using a subset of 11,177 high-quality autosomal SNPs via the –Z-genome command in PLINK [27]) was incompatible with their known pedigree relationships (and for whom such discrepancies could not be resolved on further investigation). SNP quality control checks were used to retain only a subset of the genome-wide SNPs that could be expected to be of high quality. For the current investigation, we used slightly more stringent SNP exclusion thresholds than had been used in [13], namely SNPs were excluded if their minor allele frequency was 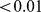, if the Fisher information for the allele frequency 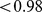, if call rate 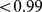, or if the p-value for a test of Hardy Weinburg Equilibrium 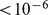. These quality control checks resulted in the retention of 1972 genotyped individuals (357 cases, 1613 controls and two individuals of unknown phenotype) from 308 families (244 from Natal, 64 from Belém), each genotyped at 545,433 autosomal SNPs.

For the majority of analyses considered here, we used either the 1972 genotyped individuals or else the entire set of 3626 individuals (with or without genotype data) that are required to define the ‘known’ (theoretical) pedigree relationships. For power comparisons between LMM methods, we also investigated use of a subset of 462 ‘founder’ individuals, chosen on the basis of theoretical relationships and estimated kinships to be approximately unrelated to one another.

### Generation of simulated phenotypes

We generated simulated phenotypes for the 1972 individuals that had genome-wide SNP data available. We used two different models for generating binary (disease) traits, one corresponding to ‘strong’ genetic effects (sim-D1) and one corresponding to ‘weak’ genetic effects (sim-D2), with the trait in both cases governed by two SNPs (rs9271252 and rs233722) located on chromosomes 6 and 12 respectively. In addition to modelling genetic effects at rs9271252 and rs233722, we allowed for 22 weaker ‘polygenic’ effects caused by genotype at the 100th SNP on each autosomal chromosome. Each effect contributed multiplicatively to the probability of developing disease. Thus, the mathematical model for generating the simulated phenotype was 
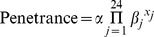
where *x_j_* was a variable coded (0, 1, 2) according to the number of copies of the risk allele possessed at causal SNP *j* (with *j* = 1 corresponding to rs9271252 and *j* = 2 corresponding to rs233722), the baseline penetrance 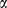 was set to equal 0.017 for the ‘strong’ scenario and 0.022 for the ‘weak’ scenario, 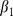 was set to equal 2 for the ‘strong’ scenario and 1.6 for the ‘weak’ scenario, 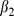 was set to equal 1.8 for the ‘strong’ scenario and 1.55 for the ‘weak’ scenario, and 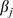 (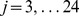) was set to equal 1.1 under both scenarios. Resulting penetrances greater than 1.0 were assigned to equal 1.0.

We also simulated a model (sim-Q) for quantitative traits, again governed by rs9271252 and rs233722 on chromosomes 6 and 12. The traits were generated as a linear combination of the effect from each of the strong and polygenic effect SNPs, with a normally distributed error component, thus: 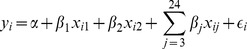
where *x_ij_* was a genotype variable for person *i* at SNP *j* coded as above, 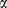 represents the baseline trait and was set to 100, 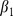 was set to 3, 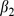 to 2, 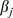 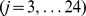 which correspond to polygenic contributions for SNP *i* were set to 1, and 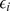 was a randomly generated variable following a normal distribution with mean 0 and standard deviation 5.

We simulated a model (sim-L20) for longitudinal quantitative traits (with *k* = 20 repeated measures for each individual) in a rather similar manner, with individuals' non-genetic variation accounted for by another error term 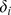: 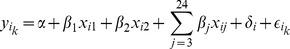



The baseline trait 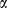 remained 100, 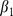 was set to 5, 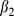 to 4, 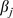 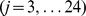 were set to 1.5, 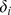 was a random variable following a normal distribution with mean 0 and standard deviation 4, generated once for each individual. The residual error term 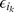 was a randomly generated variable following a normal distribution with mean 0 and standard deviation 2.

To make the analyses feasible whilst still maintaining the overall degree of relatedness, the longitudinal data set was constructed based on a subset of 498 individuals selected through stratified sampling from the original data set, with number of individuals randomly selected from each extended family approximately proportional to their family size while also ensuring that every family is represented by at least one individual. Phenotypes for these 498 individuals were then generated 20 times to create the final longitudinal data set.

In addition we simulated a purely polygenic longitudinal model (sim-P20) in which the strong effects 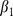 and 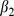 did not exist, and the 22 polygenic effects 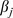 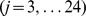 were replaced by 402 polygenic effects 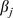 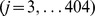 which were set to 0.75. In this model, 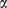 was set to 20, 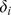 followed a normal distribution with mean 0 and standard deviation 16, and 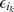 followed a normal distribution with mean 0 and standard deviation 1.

We generated 1000 replicates of each simulated data set, apart from the longitudinal and polygenic longitudinal data sets for which we only simulated a single replicate. For visualisation of results from a whole genome scan, we analysed only a single replicate (replicate 1). For investigation of power, type 1 error and concordance, to reduce computation time we analysed all 1000 replicates but only generated test statistics at 40 SNPs that lay within 40 kb of the simulated disease loci (for evaluation of power) and 20 SNPs that lay well outside the region of any simulated disease loci (for evaluation of type 1 error). By default, the programs Mendel and ROADTRIPS require all SNPs that are being used to estimate genome-wide relatedness to also be read in and tested for association; to perform the analysis of all 1000 replicates in reasonable time we therefore included the 50,129 ‘pruned’ SNPs rather than the full genome-wide set of SNPs that would normally be used by these programs.

### Linear mixed models methods and software

All the LMM implementations evaluated here attempt to fit either an exact or an approximate version of the standard linear mixed model: 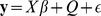
where 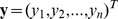 is a vector of responses (either quantitative traits or binary traits coded 1/0 for case/control status) on *n* subjects, 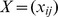 is the 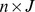 matrix of predictor variables to be modelled as fixed effects, including variables representing genetic and/or non-genetic covariates as well as a vector of variables 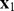 representing the genotypes at a particular SNP currently being tested (generally coded as (0,1,2) according to the number of copies of a particular allele possessed), 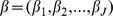 are regression coefficients (to be estimated) representing the linear effects of predictors on response, and *Q* and 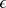 are random effects assumed to follow the distributions 
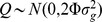
 and 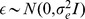 respectively (where 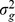 and 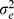 are parameters to be estimated representing genetic and environmental components of variance, *I* is the 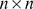 identity matrix and 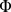 is an 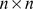 matrix of pairwise kinship coefficients).

#### GenABEL (FASTA)

The mmscore and polygenic functions of the GenABEL package [39] together allow implementation of the FAmily based Score Test Approximation (FASTA) method proposed by Chen and Abecasis [9]. The FASTA method is also implemented in the --fast-Assoc option of the MERLIN [40] package, however MERLIN calculates the kinship matrix 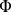 internally on the basis of known (theoretical) kinships constructed from known pedigree relationships, rather than allowing the pairwise kinship coefficients to be estimated using genome-wide SNP genotype data [12]. We therefore preferred to use GenABEL, which can read in a user-specified matrix 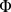 constructed on the basis of either theoretical or estimated kinship coefficients.

Rather than fitting the full linear mixed model 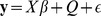 and estimating 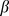, 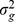 and 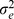 by maximum likelihood for each SNP across the genome, FASTA implements an ‘approximate’ two-stage approach. At the first stage a reduced model is fitted, where the regression coefficient 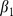 (corresponding to the effect at the SNP currently under test) is assumed to equal 0. At the second stage, a score statistic for testing the null hypothesis that 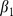 does indeed equal 0 is constructed as: 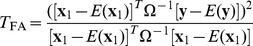
where 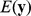 refers to an *n*-dimensional vector of fitted values of the response from the reduced model, 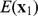 refers to an *n*-dimensional vector of unconditional expectations of genotype scores at the test SNP (each element of which equals twice the allele frequency of the particular allele being counted), and 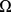 refers to the estimated variance/covariance matrix, 
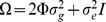
, with 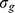 and 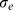 taking their maximum likelihood estimates as calculated under the reduced model. The score statistic is calculated repeatedly using the appropriate *n*-dimensional vector 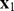 for each test SNP (typically between 500,000 and several million SNPs) across the genome, but the time-consuming maximum likelihood step for estimating 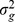, 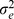 and 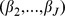 need only be performed once, at the start.

#### GenABEL (Grammar-Gamma)

The grammar function of the GenABEL package [39] implements the GRAMMAR-Gamma method proposed by Svishcheva et al. [14]. This method can be considered as an extension of the original GRAMMAR method [10], [12] to produce a test that is essentially a fast approximation to FASTA.

In GRAMMAR [10], similarly to FASTA, the first step is to fit a reduced version of the full linear mixed model in which 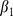 is set to 0. Phenotype residuals 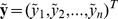 may be constructed as 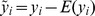 where 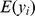 refers to the fitted value of the response for person *i* from the reduced model. These residuals are then used as the independent trait in a simple linear regression model: 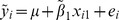
where the error term *e_i_* is assumed to be independently normally distributed. Estimation of 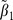 and testing of the null hypothesis that 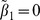 can be accomplished through maximum likelihood or least squares approaches. Alternatively, a rapid test of 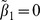 can be achieved [12], [14] through construction of a score statistic: 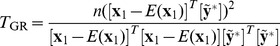
where 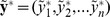 are transformed version of the residuals 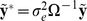. Again, the time-consuming maximum likelihood step for estimating 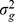, 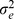 and 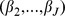 (and thus for calculating the transformed residuals 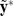) need only be performed once.

In the original GRAMMAR publication [10], the assumption was that pedigree relationships between individuals would be known and so 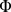 would be constructed on the basis of theoretical kinship coefficients. Subsequently it was suggested [12] that the use of estimated kinship coefficients (estimated on the basis of genome-wide SNP data) could perform as well or better. Regardless of which kinship coefficients are used, GRAMMAR was found to be conservative and to result in biased regression coefficients representing the SNP effects of interest [12], and so it was suggested that the final 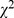 test statistics should be ‘re-inflated’ by multiplying by an appropriate estimated correction factor (in a procedure analogous to the ‘deflation’ of 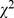 test statistics via genomic control [29]) to result in a final test statistic with the appropriate null distribution. This ‘genomic control corrected’ version of GRAMMAR was denoted GRAMMAR-GC by [12].

The GRAMMAR-Gamma method [14] is similar to GRAMMAR but, unlike GRAMMAR, produces unbiased SNP effect estimates and test statistics that do not require any deflation. The method involves calculating a GRAMMAR-Gamma correction factor 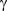 (see [14] for details) that is used to adjust a new statistic 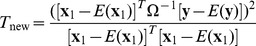
which can be calculated from a standard linear regression analysis of 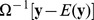 on 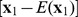. This results in a final GRAMMAR-Gamma statistic *T*_GRG_ = *T*_new_/γ that can be shown to be approximately equivalent to the FASTA statistic 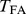. Svishcheva et al. [14] argue that their GRAMMAR-Gamma method has similar computational complexity to alternative methods such as FASTA, EMMAX and FaST-LMM at stage 1, while achieving computational savings over these methods at stage 2 (achieving a stage 2 computational complexity of *O*(*sn*), where *n* is the sample size and *s* the number of SNPs to be tested).

#### EMMAX

Kang et al. [1] proposed a method that appears to be essentially equivalent to the FASTA method proposed by Chen and Abecasis [9], except for the following caveats:

1. In the approach of Kang et al. [1], there is no expectation that the individuals will be closely related, indeed the method is motivated as an alternative to principal component based approaches when adjusting for population substructure in genome-wide association studies of unrelated individuals. Thus, the kinship coefficients used to construct 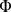 are not based on any ‘known’ pedigree relationships but are estimated based on genome-wide SNP data (using either a simple estimate based on the proportion of alleles identical-by-state (IBS) measure, or else an estimate that Kang et al. [1] describe as a Balding-Nichols (BN) estimate), resulting in a procedure essentially identical to that proposed by Amin et al. [12].
2. In the approach of Kang et al. [1], rather than applying the method solely to quantitative traits as had been done previously [9], [10], [12], the method is also proposed to apply to case/control data (with the response coded as 0 or 1, but analysed as if it were, in fact, a quantitative trait, i.e. assuming a normally distributed random environmental/error term 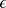). Kang et al. argue that this is computationally more convenient than fitting a generalized linear mixed model with a logit or probit link function (which would be the usual way to analyse binary response data) and should not result in increased type 1 error for testing the null hypothesis.
3. Although not entirely clear from the description in Kang et al. [1], it appears that, at the second stage, in contrast to [9], any covariates other than the SNP currently under test are re-estimated i.e. the entire vector of fixed effect predictors 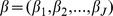 is estimated, rather than fixing 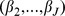 at their estimated values from the first stage.

The method of Kang et al. [1] has been implemented in the software package EMMAX. As pointed out by Lippert et al. [4], EMMAX, along with its predecessor EMMA [41], achieves additional computational efficiency (over and above that achieved by simply estimating parameters 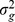 and 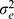 only once) by reparameterising the likelihood in terms of a parameter 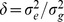 (which is estimated only once) and by making clever use of spectral decompositions. This results in a computational complexity of 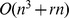 at stage 1 (where *r* the number of iterations i.e. the number of evaluations of the likelihood required) together with a computational complexity of 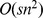 at stage 2, resulting in a total computational complexity of 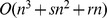.

A similar approach to [1] and [9] was proposed by Zhang et al. [2] and implemented in a software package TASSEL. The main focus of the paper by Zhang et al [2] was to describe a clustering algorithm that results in an approximation to the kinship matrix with lower effective dimensionality, which can be used in place of the full known or estimated kinship matrix. Similarly to EMMAX, in TASSEL the values of 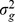 and 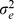 (as well as a cluster membership variable *C*) are estimated under the null hypothesis that 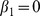 (at stage 1) and are then held fixed while estimating 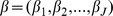 (at stage 2). The motivation for the clustering approximation is to reduce computation time. However, existing software packages (e.g. EMMAX and the mmscore and polygenic functions in GenABEL) that address the problem without making such an approximation are not computationally prohibitively time consuming. Therefore it is unclear why use of this approximation should be preferred. For this reason, given the extreme similarity between the methods implemented in EMMAX and TASSEL when no clustering is performed, we have not included TASSEL in our comparisons.

#### FaST-LMM

Lippert et al. [4] developed a fast ‘exact’ LMM implementation that, in common with EMMAX, reparameterises the likelihood in terms of a parameter 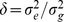, and also requires only a single spectral decomposition at the first stage of the algorithm, resulting in a total time complexity of 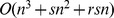. This exact method is the default in the current (2.04) version of FaST-LMM. (In previous versions the default was to use an approximate method in which 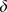 is fixed to its value from fitting a null model containing no fixed SNP effects, as is done in EMMAX, TASSEL and FASTA, resulting in a reduced complexity of 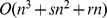. This approximate method is now available in FaST-LMM as an optional alternative to the exact method). A further speed-up can be achieved in FaST-LMM by restricting the number of SNPs used to construct the kinship matrix 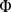 to a number less than the number of individuals.

FaST-LMM uses either maximum likelihood (ML) or restricted maximum likelihood (REML). In early versions of FaST-LMM the default was ML but in later versions the default became REML. After some experimentation, we deemed ML to be the most reliable and have used that for all results presented here.

#### GEMMA

Zhou and Stephens [16] implemented an exact approach extremely similar to that of FaST-LMM in their package GEMMA. Indeed, Zhou and Stephens themselves point out that GEMMA should give essentially identical inference to FaST-LMM in the same time complexity 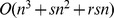, but note that the number of iterations (*r*) required to reach convergence in GEMMA is expected to be slightly smaller than in FaST-LMM, owing to the use of a more efficient optimization method. GEMMA also has an attractive practical advantage of allowing the input of imputed [42] genotype data, rather than real measured genotype data, if desired.

#### MMM

Pirinen et al. [15] have implemented approximate and exact approaches similar to the approximate and exact approaches of FaST-LMM (and the exact approach of GEMMA) in their package MMM. An advantage of MMM in comparison to the other packages is that it allows the output of regression coefficients and standard errors for the SNP effects on the (log) odds ratio scale, making it convenient to compare or combine the results with results from traditional case/control studies analysed via logistic regression. In addition, MMM allows the input of imputed genotype data rather than real measured genotype data, if desired. MMM was used in the original analysis of the Brazilian VL family data described in [13]. For more details on the methodology implemented in MMM, see [15].

#### Mendel

An approximate (score test) LMM implementation, suitable for analysis of GWAS data, has also been implemented in the software package Mendel [35] (versions 13.0 and higher). A slower (exact) LMM implementation is also available, but we only considered the approximate test here. Mendel can a. calculate kinship coefficients on the basis of known pedigree relationships, b. use the full set of genome-wide SNP data to cluster people into apparent pedigrees and then estimate kinship coefficients within those pedigree clusters, or c. use kinship coefficients estimated for all pairs of genotyped individuals on the basis of their full set of genome-wide SNPs. The resulting tests should be conceptually extremely similar to the LMM tests implemented in other software packages such as EMMAX and FaST-LMM.

### Alternative methods and software

#### FBAT

Traditional approaches for family-based association analysis focus on the transmission of high-risk alleles through pedigrees, in an approach that is closely related to traditional linkage analysis. Indeed, the well-known transmission disequilibrium test (TDT) [19], which tests whether a particular allele is transmitted preferentially from heterozygous parents to affected offspring, was originally developed as a test of linkage in the presence of association, rather than as a test of association per se. In this context, by ‘linkage’ we mean the transmission from parent to offspring of alleles in coupling at a test (marker) locus and an unobserved causal locus, i.e. the phenomenon whereby alleles that are in coupling (on the same haplotype) in the parent tend to be transmitted together to the offspring, whereas by ‘association’ we mean population-level correlation between alleles at the two loci (usually referred to as linkage disequilibrium (LD)), i.e. the tendency for alleles at the two loci to occur in coupling in the founders of a pedigree.

The TDT was originally designed for the analysis of case/parent trios (i.e. units consisting of an affected child together with their parents) but has been extended to allow analysis of nuclear families and larger pedigrees [20], [21], [23], [43]–[46]. Here we focus on the family-based association test (FBAT) [21], [23], as implemented in the FBAT software package. FBAT can be thought of as a general class of test statistics of the form 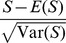
where 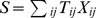 and *X_ij_* is some genotype variable and *T_ij_* some trait variable for offspring *i* in nuclear family *j*. The exact form of FBAT thus depends on the genotype and trait coding used. Genotype is generally coded in allelic fashion with a variable coded (0, 1, 2) according to the number of copies of the high-risk allele possessed. The trait variable is constructed as 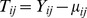 where Y*_ij_* is coded 0/1 (for binary traits such as disease status) and 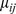 is an offset that can be chosen to consider transmissions to affected offspring only (the default), or else to contrast transmissions to affected offspring with transmissions to unaffected offspring, either weighted equally (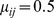) or with 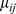 chosen to minimize the variance of test statistic. For quantitative traits, Y*_ij_* would generally correspond to the measured trait for offspring *i* in nuclear family *j*, with 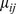 set to equal the mean trait value or else chosen to minimize the variance of test statistic.

Although, for binary traits, contrasting transmissions to affecteds with transmissions to unaffecteds seems an attractive idea, in practice this results in comparing the probability of transmission of high-risk alleles to affected individuals (which is expected, under the alternative hypothesis, to exceed 0.5) with an *estimate* of the probability of transmission of high-risk alleles to unaffected individuals (which is expected, under both null and alternative hypotheses, to approximately equal 0.5, unless the effect of the risk allele is large), rather than comparing the transmission probability to affecteds with an assumed fixed value of 0.5. For complex diseases, where the effects of risk alleles are likely to be modest (allelic odds ratios in the order 1.2–1.5), this means that greater power would be expected from the default offset that considers transmissions to affected offspring only, without paying a penalty for (imperfect) estimation of the expected 0.5 transmission probability (along with a measure of uncertainty in the estimate) from the data at hand.

By default, FBAT divides larger pedigrees into nuclear families and constructs a test that corresponds to testing ‘linkage in the presence of association’ [23]. The ‘-e’ option in FBAT allows the alternative construction of a test for ‘association in the presence of linkage’ [22], through use of an empirical variance/covariance estimator that adjusts for the correlation among sibling genotypes and for different nuclear families within a single pedigree. Use of the ‘-e’ option is expected to give smaller test statistics (larger p-values) than the default analysis, since it accounts for the fact that the effective sample size is smaller when considering FBAT as a test of association than as a test of linkage. Since, for complex diseases, we are interested in maximizing the power for detection of an effect, rather than in ensuring that the detection is genuinely driven by association (rather than linkage) between alleles at our test locus and the underlying unobserved causal locus, we use the default option in all analyses presented here. From a practical point of view, this means that any signal we detect may in fact be marking a true effect that lies some distance away, rather than necessarily being located in the immediate vicinity of the detected signal.

#### ROADTRIPS and MQLS

Thornton and McPeek [26] implemented a ‘**RO**bust **A**ssociation- **D**etection **T**est for **R**elated **I**ndividuals with **P**opulation **S**ubstructure’ in a package called ROADTRIPS. ROADTRIPS can be thought of as an extension of their previously-proposed Maximum Quasi-Likelihood Statistic (MQLS) [24]. Both MQLS and ROADTRIPS construct adjusted versions of standard case/control 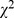 (or Armitage Trend) tests, adjusting for the known relatedness between individuals (that would ordinarily cause an inflation in standard case/control tests) through a kinship matrix that models the known pedigree relationships. ROADTRIPS (but not MQLS) additionally makes use of a covariance matrix based on estimated kinships (as estimated from genome-wide SNP data) to further correct for additional unknown relatedness and population stratification.

The ROADTRIPS test statistic takes the form: 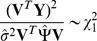



Thornton and McPeek note that many commonly-used case/control statistics can be coerced into this form. Here 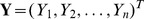 is genotype vector at a test SNP for *n* individuals (coded using an allelic coding), **V** is a vector of length *n* coding for phenotype information (disease status) and known (or externally estimated) relationships (see [26] for details of its construction), 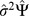 is an estimate of the null variance/covariance matrix of **Y** (so that 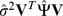 is an estimate of null variance/covariance of 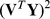), 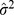 is an estimate of Var(**Y**) in an outbred population and 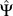 is an internally estimated matrix used to simultaneously adjust for unknown relatedness/pedigree relationship errors and population stratification.

#### MASTOR and GTAM

Recently, Jakobsdottir and McPeek [25] proposed a retrospective approach (MASTOR) for analysis of quantitative traits that can be considered essentially as a quantitative trait version of MQLS. In common with MQLS, kinships are assumed to be estimated on the basis of known pedigree relationships, but in principle kinships estimated from genome-wide SNP data could be read in instead. Jakobsdottir and McPeek compared MASTOR to a previously-proposed LMM method, GTAM [8], and found MASTOR to have some advantages. The main advantage of MASTOR over GTAM (and many other approaches) is that, in common with MQLS and ROADTRIPS, MASTOR allows information to be gained from individuals who are phenotyped but not genotyped. Both MASTOR and GTAM are implemented within the MASTOR software package. Although designed for analysis of quantitative (rather than binary) traits, given that the spirit of recent LMM approaches has been to apply approaches originally designed for quantitative traits to binary traits (coded as 0 and 1), we investigated the performance of MASTOR and GTAM when applied to both binary and quantitative traits.

### Calculation of kinship coefficients

The LMM approaches considered here, as well as methods such as MQLS, ROADTRIPS, MASTOR and GTAM, all involve modelling the relatedness between individuals through one or more kinship matrices, constructed either on the basis of known (hypothesized) pedigree relationships between individuals, or through estimating kinships on the basis of genome-wide SNP data (or from a subset of available genome-wide SNPs). The precise algorithms used to estimate kinships on the basis of genome-wide SNP data vary [36], [37], [47], although we have found the kinship matrices from the different packages we considered to be largely comparable (see Results). Most packages allow a separation between the estimation of the kinship matrix step and the analysis (incorporating the desired kinship matrix) step. This is convenient as it allows a potentially different set of SNPs to be used for estimating the kinship matrix as is used for genome-wide association testing. It also means that kinships estimated using one package can potentially be read in to another package at the analysis stage, if desired. For the majority of analyses performed here, we used the same software package (or a recommended accompanying software package) to calculate the kinship matrix as we used for subsequent association testing, and to estimate the kinship matrix we used a subset of 50,129 ‘pruned’ SNPs with minor allele frequencies 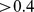 and ‘pruned’ to be in approximate linkage equilibrium via the - -indep 50 5 2 command in PLINK [27]). (We found little difference between the results obtained when using such a pruned set of SNPs and using the full genome-wide set of SNPs, see Results).

We also explored the use of a smaller set of 1900 ‘thinned’ SNPs to estimate kinships. This number was chosen to capitalise on the speed-up that can be achieved in FaST-LMM by restricting the number of SNPs used to construct the kinship matrix 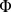 to a number less than the number of individuals. The ‘thinned’ SNPs comprised an evenly-spaced subset of the ‘pruned’ SNPs selected based purely on physical position using the software package MapThin (http://www.staff.ncl.ac.uk/richard.howey/mapthin/). In addition we explored the use of the FaST-LMM-Select procedure [30], implemented within the FaST-LMM package, that uses an iterative procedure to select SNPs for inclusion in the construction of the kinship matrix on the basis of their nominal association with phenotype (as evaluated through a fixed effects linear regression analysis). However, we did not find this procedure to be superior to using either the pruned or the full set of SNPs (see Results).

Several alternative packages exist for estimating genetic relationships from genome-wide SNP data, either for subsequent use in LMM type analyses [48] or in order to infer pedigree relationships as an end in itself [28]. We investigated use of the kinship estimates output by the packages PLINK [27] and KING [28], in comparison to those calculated internally by the various LMM packages we had used. Another popular package is GCTA [48]; we note that the realised relationship matrix (RRM) kinship estimation approach used internally by FaST-LMM is theoretically equivalent to that used by GCTA.

## Supporting Information

Figure S1Comparison of estimated kinship measures and −log10(p-values) obtained based on full, pruned and thinned SNPs. (A) Estimated kinship measures (B) 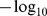 p-values obtained. F =  full set, P =  pruned set, T =  thinned set. EM_BN = EMMAX (Balding-Nichols), EM_IBS = EMMAX (IBS method), FLMM_C = FaST-LMM using covariance matrix, FLMM_R = FaST-LMM using realised relationship matrix, GA = GenABEL, GA_FA = GenABEL (FASTA), GA_GRG = GenABEL (GRAMMAR-Gamma), GMA_C = GEMMA using centred genotypes, GMA_S = GEMMA using standardised genotypes, KING_H = KING with homogeneous population assumption, KING_R = KING with robust estimation, MMM_E = MMM using full mixed model (exact) calculation, MMM_G = MMM using GLS approximation.(TIF)

Figure S2 QQ plots of real VL phenotype GWAS results, using different LMM software packages and different SNP sets for kinship estimation. The black diagonal lines represent the line of equality. The “theoretical” set used pedigree structure to derive theoretical kinship coefficients. EM_BN = EMMAX (Balding-Nichols), EM_IBS = EMMAX (IBS method), FLMM_C = FaST-LMM using covariance matrix, FLMM_R = FaST-LMM using realised relationship matrix, GA_FA = GenABEL (FASTA), GA_GRG = GenABEL (GRAMMAR-Gamma), GMA_C = GEMMA using centred genotypes, GMA_S = GEMMA using standardised genotypes, MMM_E = MMM using full mixed model (exact) calculation, MMM_G = MMM using GLS approximation, Unadj  =  unadjusted analysis. For methods with two ways to estimate the kinships, the same “theoretical” results were plotted twice. Unadjusted analysis results were plotted once in each column only for comparison, and did not use the kinship estimates for adjustment.
(TIF)

Figure S3Performance of FaST-LMM-Select. Genomic control factor (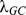) achieved in analysis of the real disease phenotype as different numbers of ordered SNPs are added in when calculating the kinship matrix ( = realised relationship matrix, RRM). Method implemented manually in FaST-LMM v2.0.(TIF)

Figure S4 Manhattan plots for real and simulated data sets using FaST-LMM. The points marked in red denote either the confirmed significant region from Fakiola et al. (2013) (real phenotype), or the regions close to the simulated strong/weak effect SNPs (simulated phenotypes). real  =  real VL phenotype, sim-D1 =  simulated strong binary (disease) trait, sim-D2 =  simulated weak binary (disease) trait, sim-Q =  simulated quantitative trait, sim-L20 =  simulated longitudinal quantitative trait with 20 observations, sim-P20 =  simulated polygenic longitudinal quantitative trait with 20 observations.
(TIF)

Figure S5 Manhattan plots for the simulated weak binary (disease) phenotype using FaST-LMM exact and alternative software packages. The points marked in red denote the regions close to the simulated weak effect SNPs. FLMM_E = FaST-LMM using exact calculation, RT = ROADTRIPS, FBATaff = FBAT using transmissions to affecteds only, FBATboth = FBAT using transmissions to both affecteds and unaffecteds. Results from all other LMM methods were indistinguishable from FLMM_E and so are not shown. MQLS and RT gave identical results with either 1972 or 3626 individuals, as phenotypes could only be simulated for the 1972 genotyped individuals.
(TIF)

Figure S6 Manhattan plots for the simulated strong binary (disease) phenotype using FaST-LMM exact and alternative software packages. The points marked in red denote the regions close to the simulated weak effect SNPs. FLMM_E = FaST-LMM using exact calculation, RT = ROADTRIPS, FBATaff = FBAT using transmissions to affecteds only, FBATboth = FBAT using transmissions to both affecteds and unaffecteds. Results from all other LMM methods were indistinguishable from FLMM_E and so are not shown. MQLS and RT gave identical results with either 1972 or 3626 individuals, as phenotypes could only be simulated for the 1972 genotyped individuals.
(TIF)

Figure S7 Comparison of −log10(p-values) using different LMM software packages, real disease phenotypes. Plots above the diagonal show a comparison of −log10(p-values), with correlations between the -log10(p-values) indicated below the diagonal. The grey solid lines represents the line of equality; the black dashed lines the linear regression line of the variable on the y axis on the variable on the x axis. EM_BN = EMMAX (Balding-Nichols), EM_IBS = EMMAX (IBS method), FLMM_A = FaST-LMM using approximate calculation, FLMM_E = FaST-LMM using exact calculation, GA_FA = GenABEL (FASTA), GA_GRG = GenABEL (GRAMMAR-Gamma), GMA_C = GEMMA using centred genotypes, GMA_S = GEMMA using standardised genotypes, MMM_E = MMM using full mixed model (exact) calculation, MMM_G = MMM using GLS approximation, Unadj  =  unadjusted analysis.
(TIF)

Figure S8 Comparison of −log(p-values) using LMM and alternative software packages, real disease phenotypes. Plots above the diagonal show a comparison of −log10(p-values), with correlations between the −log10(p-values) indicated below the diagonal. The grey solid lines represent the line of equality; the black dashed lines the linear regression line of the variable on the y axis on the variable on the x axis. FLMM_E = FaST-LMM using exact calculation, MQLS1972 = MQLS using 1972 genotyped individuals, MQLS3626 = MQLS using all 3626 individuals with or without genotype data, RT1972 = ROADTRIPS using 1972 genotyped individuals, RT3626 = ROADTRIPS using all 3626 individuals with or without genotype data, FBATaff = FBAT using transmissions to affecteds only, FBATboth = FBAT using transmissions to both affecteds and unaffecteds, MQLS_E = MQLS using estimated (rather than theoretical) kinships.
(TIF)

Figure S9 Comparison of −log(p-values) using LMM and alternative software packages, simulated weak binary (disease) phenotype. Plots above the diagonal show a comparison of –log10(p-values), with correlations between the –log10(p-values) indicated below the diagonal. The grey solid lines represent the line of equality; the black dashed lines the linear regression line of the variable on the y axis on the variable on the x axis. The colours denote: red  =  the two weak effect SNPs, magenta = SNPs within 500 kb of the weak effect SNPs, blue  = 22 polygenic SNPs, green  = SNPs within 500 kb of the polygenic SNPs, black  =  all other SNPs. Because the black/green/blue SNPs were plotted before the magenta/red SNPs, they may be obscured by the latter. FLMM_E = FaST-LMM using exact calculation, MQLS = MQLS using 1972 or 3626 individuals, RT = ROADTRIPS using 1972 or 3626 individuals, FBATaff = FBAT using transmissions to affecteds only, FBATboth = FBAT using transmissions to both affecteds and unaffecteds. MQLS and RT gave identical results with either 1972 or 3626 individuals, as phenotypes could only be simulated for the 1972 genotyped individuals.
(TIF)

Figure S10 Comparison of −log10(p-values) obtained from FaST-LMM using alternative kinship estimates, real disease phenotypes. Plots above the diagonal show a comparison of –log10(p-values), with correlations between the –log10(p-values) indicated below the diagonal. The grey solid lines represents the line of equality; the black dashed lines the linear regression line of the variable on the y axis on the variable on the x axis. KING_H = KING homogeneous method, KING_R = KING robust method, Ped  =  theoretical kinship estimates based on pedigree information, FLMM_R = FaST-LMM's own realised relationship matrix, Unadj  =  unadjusted, Wrong  =  misspecified kinships, chosen to be inversely related to the true kinship value.
(TIF)

Figure S11 Power and type 1 error of different LMM methods applied to 462 Brazilian founders. Powers (left hand plots) are defined as the proportion of replicates (out of 1000) in which both simulated disease loci are detected, with ‘detection’ corresponding to any SNP within 40 kb of the simulated disease locus reaching the specified *p*-value threshold. Type 1 errors (right hand plots) are defined as the proportion of null SNPs (out of 20,000 = 20 null SNPs times 1000 simulation replicates) that reach the specified *p*-value threshold. Horizontal dashed lines indicate the target *p*-value thresholds (i.e. the expected type 1 error rates).
(TIF)

Table S1 Genomic control factors achieved in analysis of the real data, or a single replicate of the simulated data, when feeding externally estimated kinships into FaST-LMM.
(PDF)

Table S2 Computational speed and ease of use of various packages.
(PDF)

Table S3 Concordance between top SNPs identified by different LMM methods when using 462 founder individuals.
(PDF)

Text S1 Membership of Wellcome Trust Case Control Consortium 2.
(DOC)
