# Supplementary figures and images for: Comparison of Methods to Account for Relatedness in Genome-Wide Association Studies with Family-Based Data
Source: PLoS Genet. 2014 Jul 17;10(7):e1004445. doi: 10.1371/journal.pgen.1004445 (PMC4102448; doi:10.1371/journal.pgen.1004445)

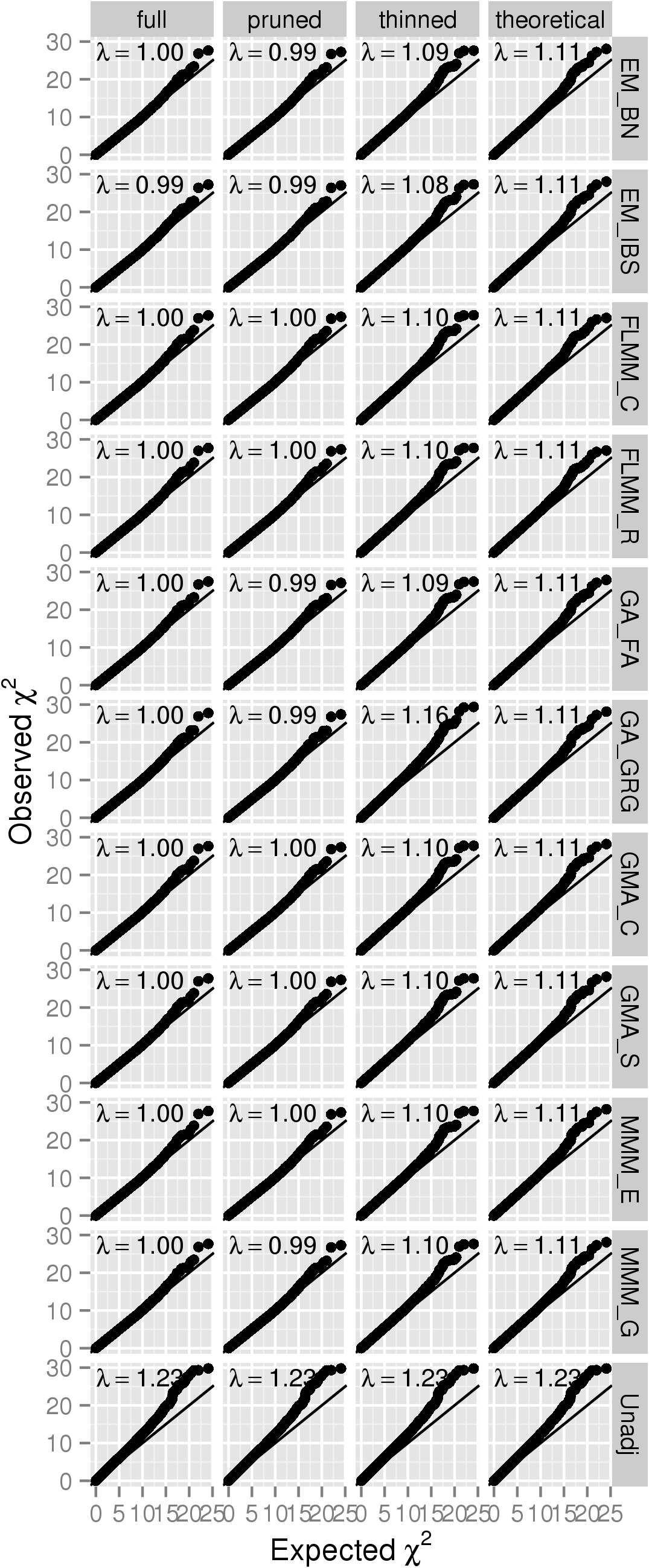

Supplement: Figure S2 — QQ plots of real VL phenotype GWAS results, using different LMM software packages and different SNP sets for kinship estimation. The black diagonal lines represent the line of equality. The “theoretical” set used pedigree structure to derive theoretical kinship coefficients. EM_BN = EMMAX (Balding-Nichols), EM_IBS = EMMAX (IBS method), FLMM_C = FaST-LMM using covariance matrix, FLMM_R = FaST-LMM using realised relationship matrix, GA_FA = GenABEL (FASTA), GA_GRG = GenABEL (GRAMMAR-Gamma), GMA_C = GEMMA using centred genotypes, GMA_S = GEMMA using standardised genotypes, MMM_E = MMM using full mixed model (exact) calculation, MMM_G = MMM using GLS approximation, Unadj = unadjusted analysis. For methods with two ways to estimate the kinships, the same “theoretical” results were plotted twice. Unadjusted analysis results were plotted once in each column only for comparison, and did not use the kinship estimates for adjustment. (TIF) [file pgen.1004445.s002.tif]

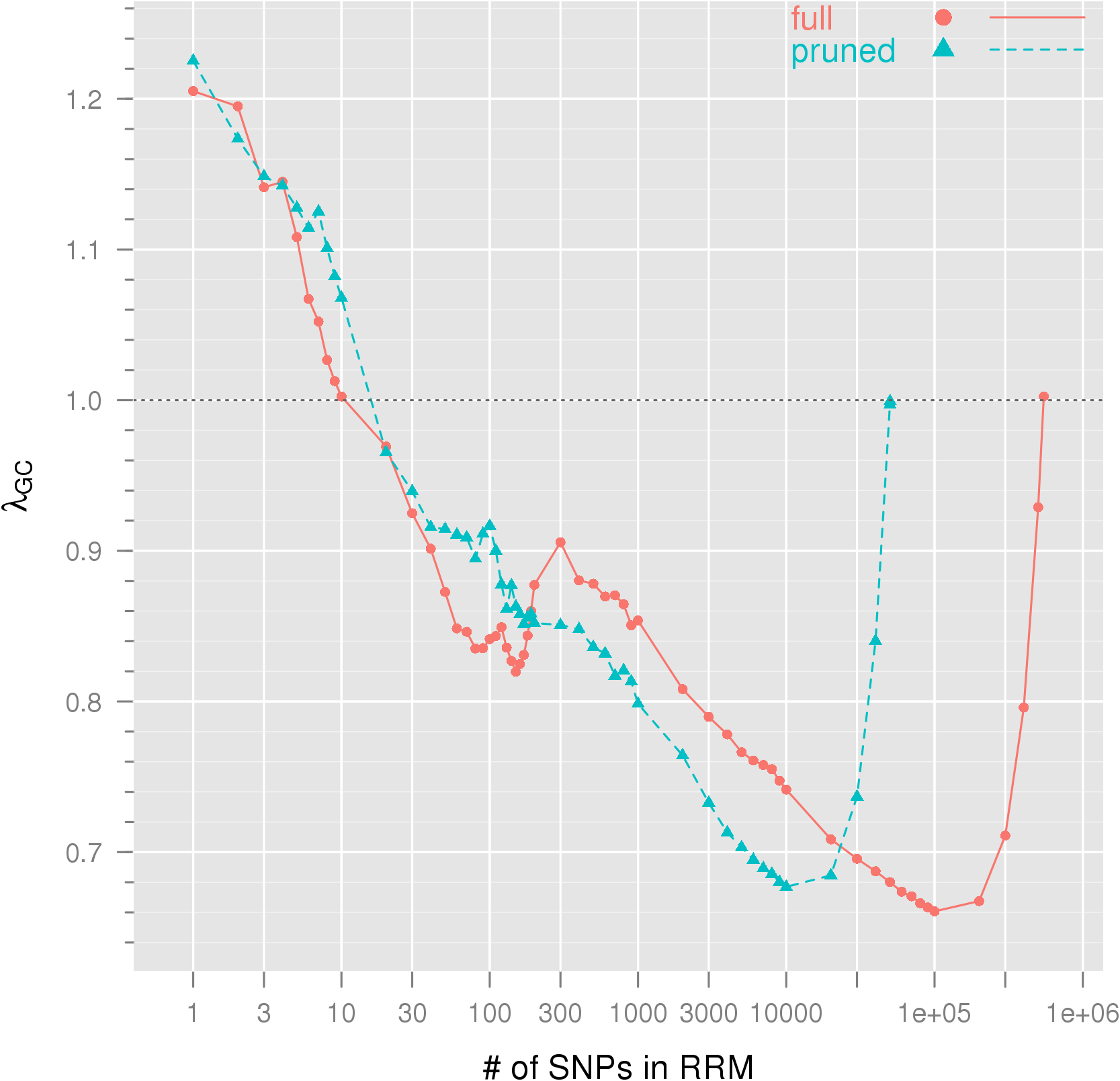

Supplement: Figure S3 — Performance of FaST-LMM-Select. Genomic control factor () achieved in analysis of the real disease phenotype as different numbers of ordered SNPs are added in when calculating the kinship matrix ( = realised relationship matrix, RRM). Method implemented manually in FaST-LMM v2.0. (TIF) [file pgen.1004445.s003.tif]

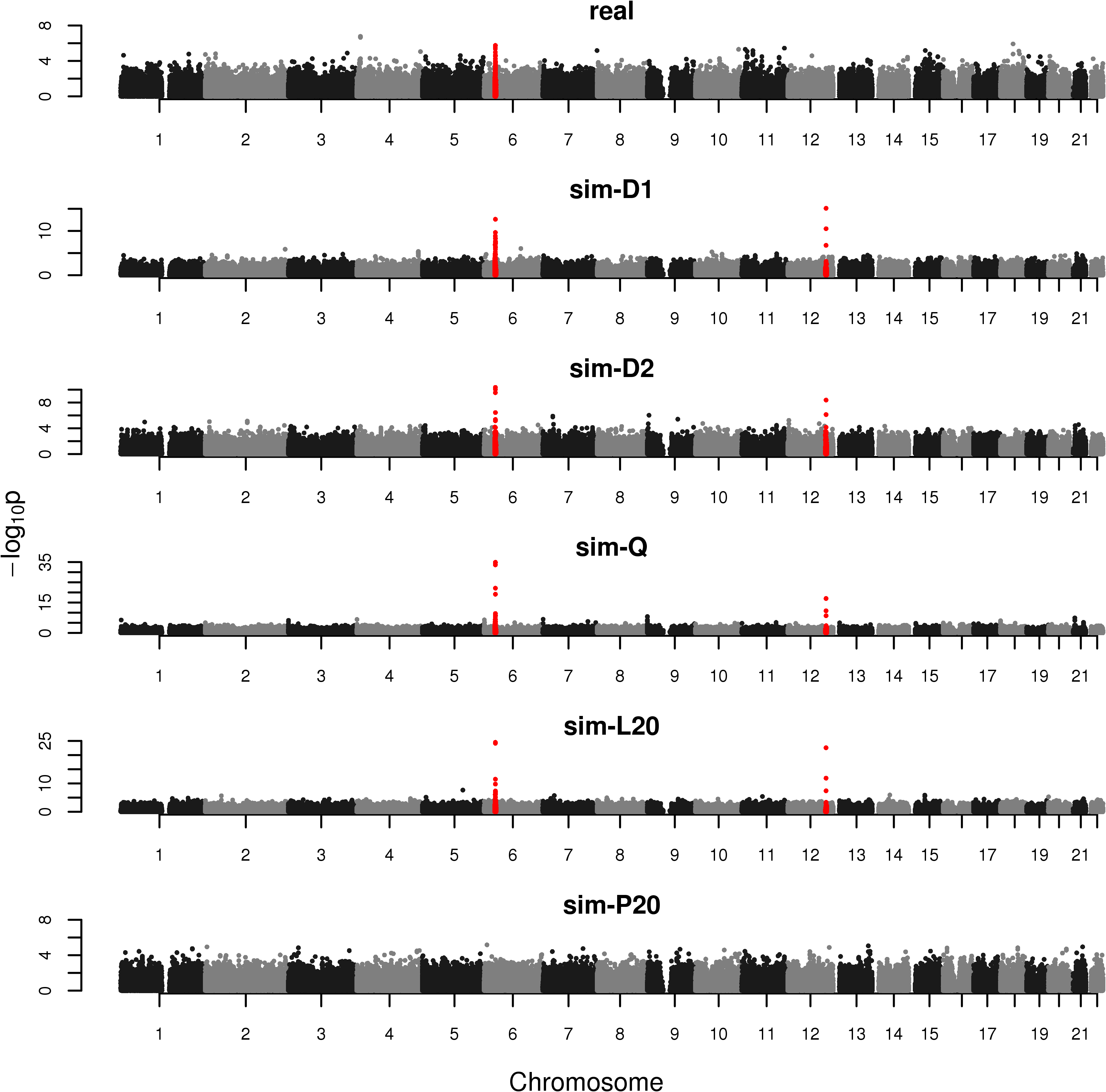

Supplement: Figure S4 — Manhattan plots for real and simulated data sets using FaST-LMM. The points marked in red denote either the confirmed significant region from Fakiola et al. (2013) (real phenotype), or the regions close to the simulated strong/weak effect SNPs (simulated phenotypes). real = real VL phenotype, sim-D1 = simulated strong binary (disease) trait, sim-D2 = simulated weak binary (disease) trait, sim-Q = simulated quantitative trait, sim-L20 = simulated longitudinal quantitative trait with 20 observations, sim-P20 = simulated polygenic longitudinal quantitative trait with 20 observations. (TIF) [file pgen.1004445.s004.tif]

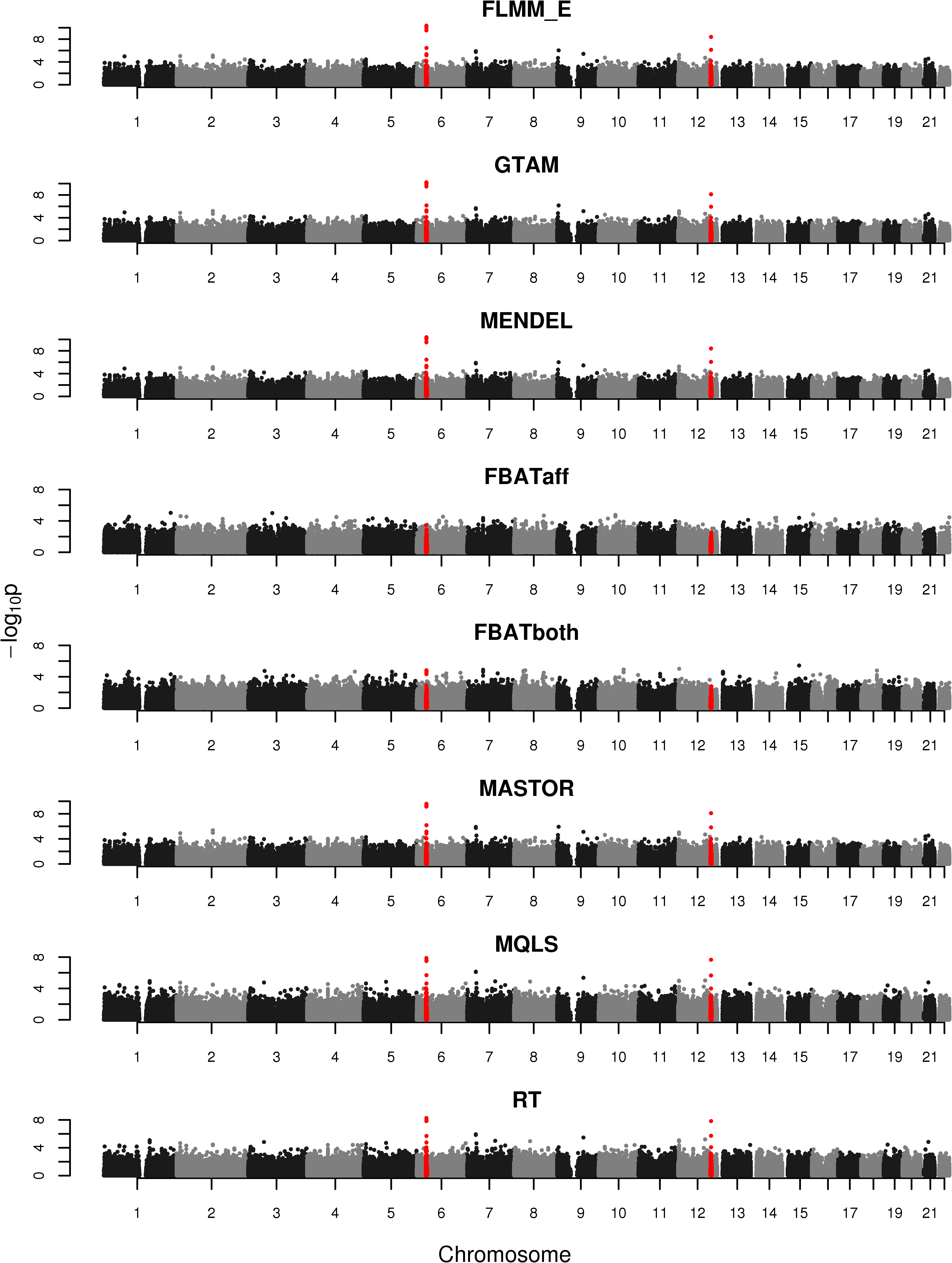

Supplement: Figure S5 — Manhattan plots for the simulated weak binary (disease) phenotype using FaST-LMM exact and alternative software packages. The points marked in red denote the regions close to the simulated weak effect SNPs. FLMM_E = FaST-LMM using exact calculation, RT = ROADTRIPS, FBATaff = FBAT using transmissions to affecteds only, FBATboth = FBAT using transmissions to both affecteds and unaffecteds. Results from all other LMM methods were indistinguishable from FLMM_E and so are not shown. MQLS and RT gave identical results with either 1972 or 3626 individuals, as phenotypes could only be simulated for the 1972 genotyped individuals. (TIF) [file pgen.1004445.s005.tif]

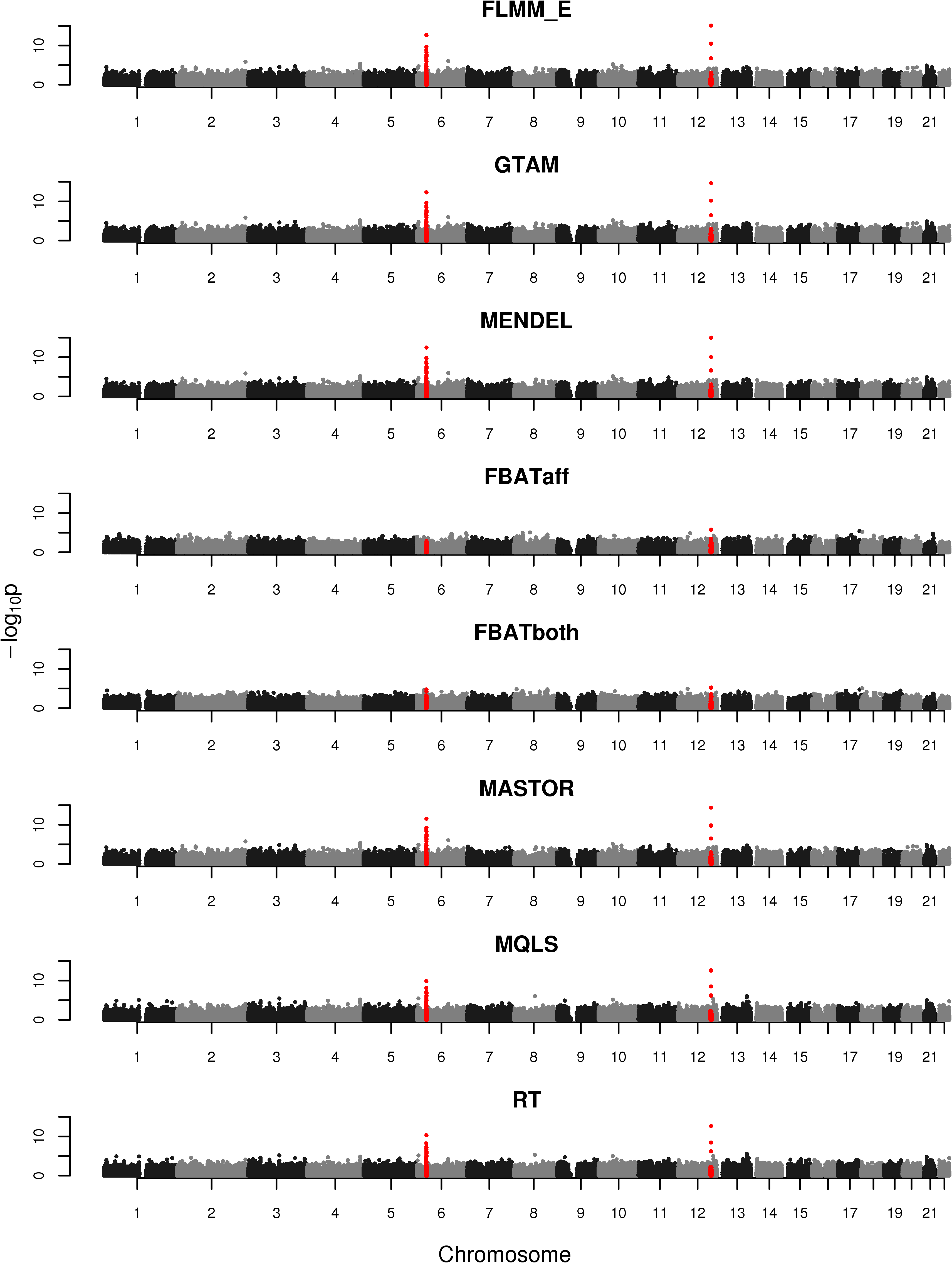

Supplement: Figure S6 — Manhattan plots for the simulated strong binary (disease) phenotype using FaST-LMM exact and alternative software packages. The points marked in red denote the regions close to the simulated weak effect SNPs. FLMM_E = FaST-LMM using exact calculation, RT = ROADTRIPS, FBATaff = FBAT using transmissions to affecteds only, FBATboth = FBAT using transmissions to both affecteds and unaffecteds. Results from all other LMM methods were indistinguishable from FLMM_E and so are not shown. MQLS and RT gave identical results with either 1972 or 3626 individuals, as phenotypes could only be simulated for the 1972 genotyped individuals. (TIF) [file pgen.1004445.s006.tif]

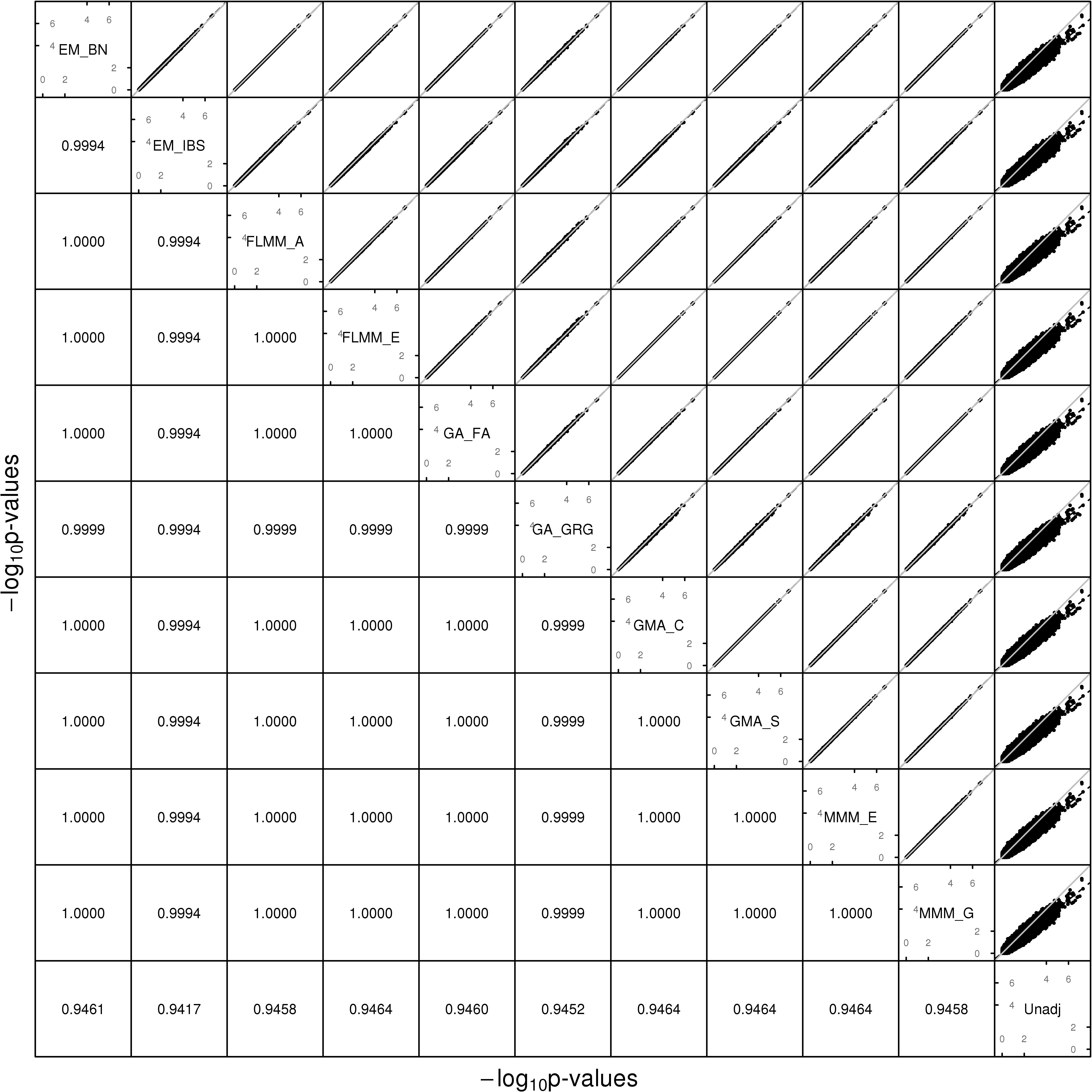

Supplement: Figure S7 — Comparison of −log10(p-values) using different LMM software packages, real disease phenotypes. Plots above the diagonal show a comparison of −log10(p-values), with correlations between the -log10(p-values) indicated below the diagonal. The grey solid lines represents the line of equality; the black dashed lines the linear regression line of the variable on the y axis on the variable on the x axis. EM_BN = EMMAX (Balding-Nichols), EM_IBS = EMMAX (IBS method), FLMM_A = FaST-LMM using approximate calculation, FLMM_E = FaST-LMM using exact calculation, GA_FA = GenABEL (FASTA), GA_GRG = GenABEL (GRAMMAR-Gamma), GMA_C = GEMMA using centred genotypes, GMA_S = GEMMA using standardised genotypes, MMM_E = MMM using full mixed model (exact) calculation, MMM_G = MMM using GLS approximation, Unadj = unadjusted analysis. (TIF) [file pgen.1004445.s007.tif]

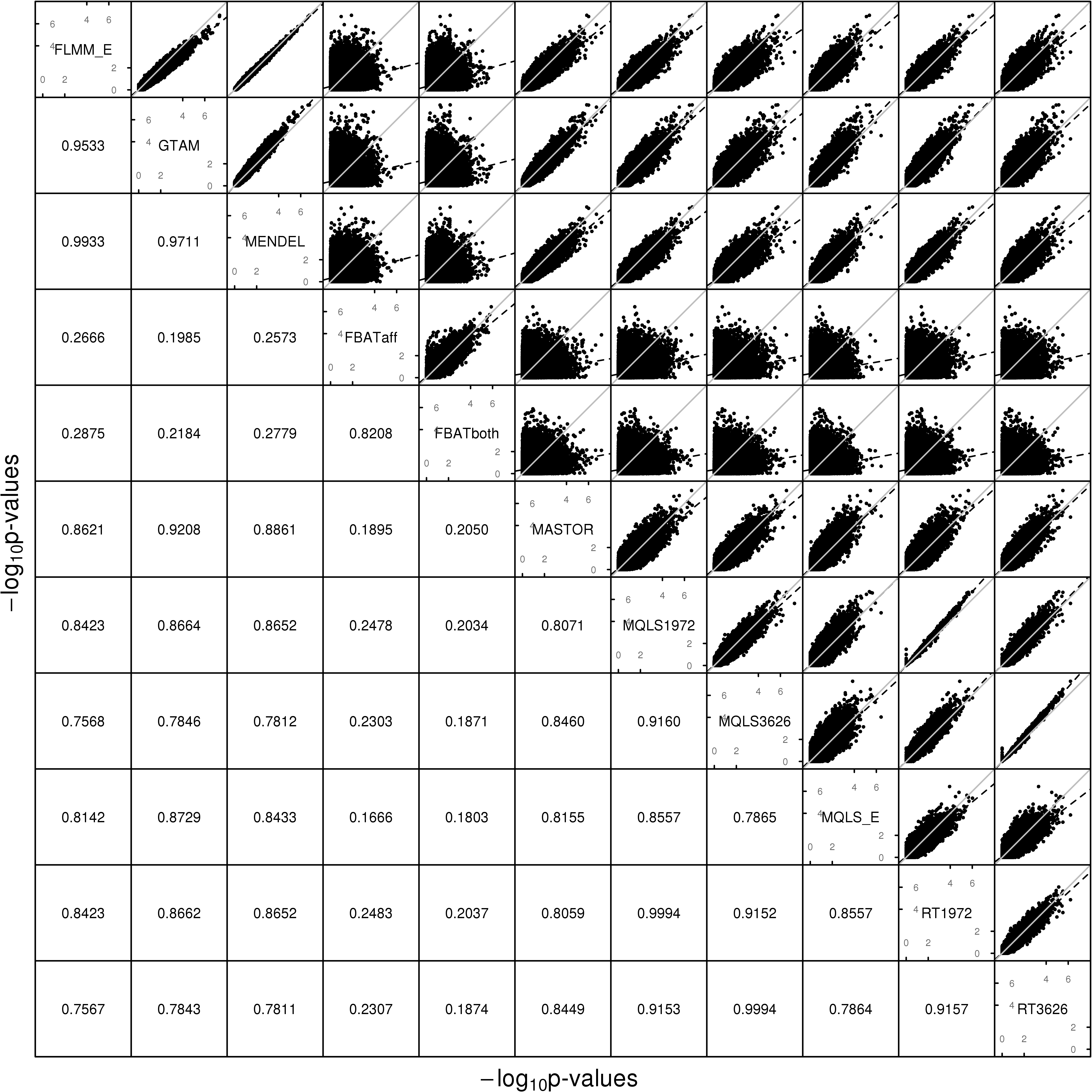

Supplement: Figure S8 — Comparison of −log(p-values) using LMM and alternative software packages, real disease phenotypes. Plots above the diagonal show a comparison of −log10(p-values), with correlations between the −log10(p-values) indicated below the diagonal. The grey solid lines represent the line of equality; the black dashed lines the linear regression line of the variable on the y axis on the variable on the x axis. FLMM_E = FaST-LMM using exact calculation, MQLS1972 = MQLS using 1972 genotyped individuals, MQLS3626 = MQLS using all 3626 individuals with or without genotype data, RT1972 = ROADTRIPS using 1972 genotyped individuals, RT3626 = ROADTRIPS using all 3626 individuals with or without genotype data, FBATaff = FBAT using transmissions to affecteds only, FBATboth = FBAT using transmissions to both affecteds and unaffecteds, MQLS_E = MQLS using estimated (rather than theoretical) kinships. (TIF) [file pgen.1004445.s008.tif]

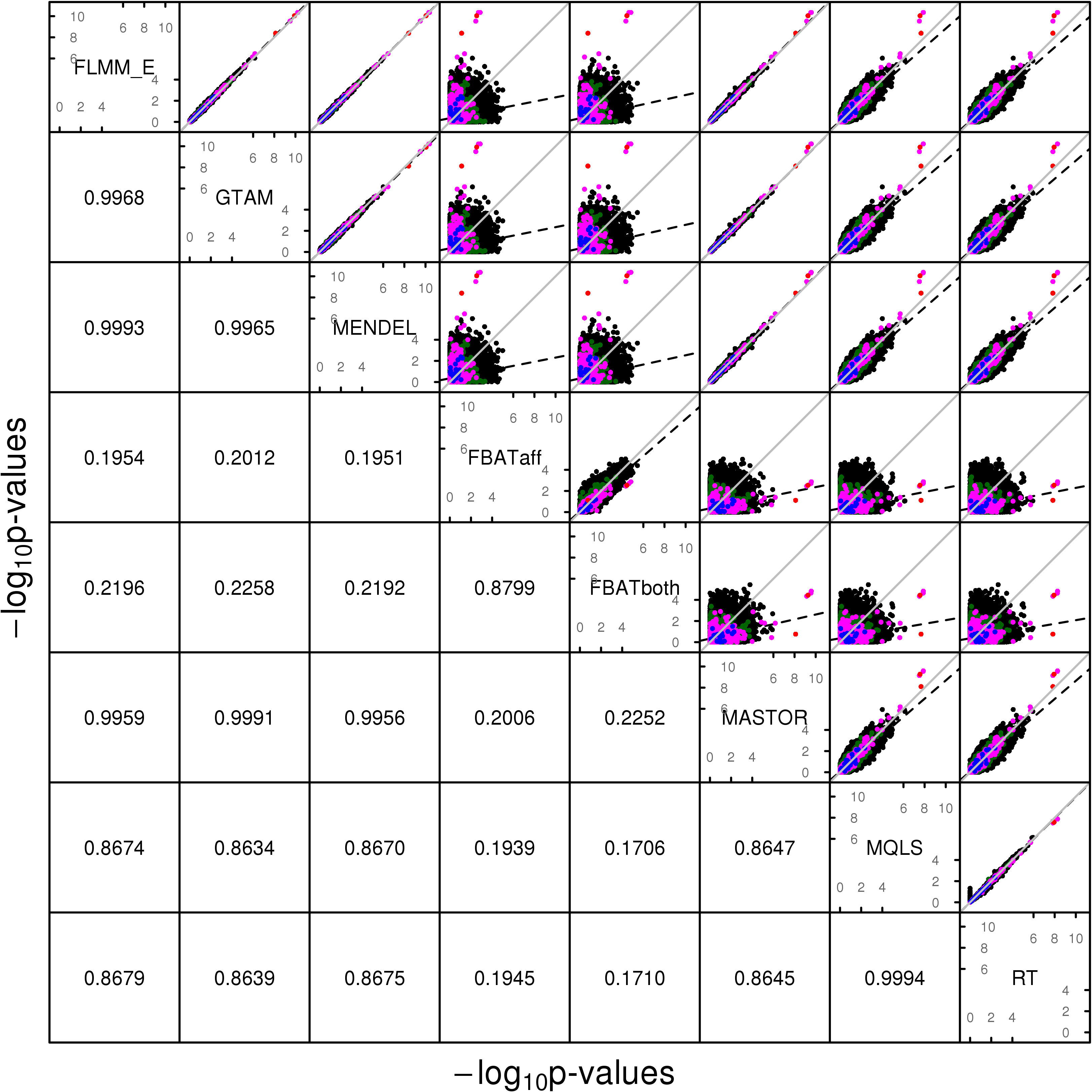

Supplement: Figure S9 — Comparison of −log(p-values) using LMM and alternative software packages, simulated weak binary (disease) phenotype. Plots above the diagonal show a comparison of –log10(p-values), with correlations between the –log10(p-values) indicated below the diagonal. The grey solid lines represent the line of equality; the black dashed lines the linear regression line of the variable on the y axis on the variable on the x axis. The colours denote: red = the two weak effect SNPs, magenta = SNPs within 500 kb of the weak effect SNPs, blue = 22 polygenic SNPs, green = SNPs within 500 kb of the polygenic SNPs, black = all other SNPs. Because the black/green/blue SNPs were plotted before the magenta/red SNPs, they may be obscured by the latter. FLMM_E = FaST-LMM using exact calculation, MQLS = MQLS using 1972 or 3626 individuals, RT = ROADTRIPS using 1972 or 3626 individuals, FBATaff = FBAT using transmissions to affecteds only, FBATboth = FBAT using transmissions to both affecteds and unaffecteds. MQLS and RT gave identical results with either 1972 or 3626 individuals, as phenotypes could only be simulated for the 1972 genotyped individuals. (TIF) [file pgen.1004445.s009.tif]

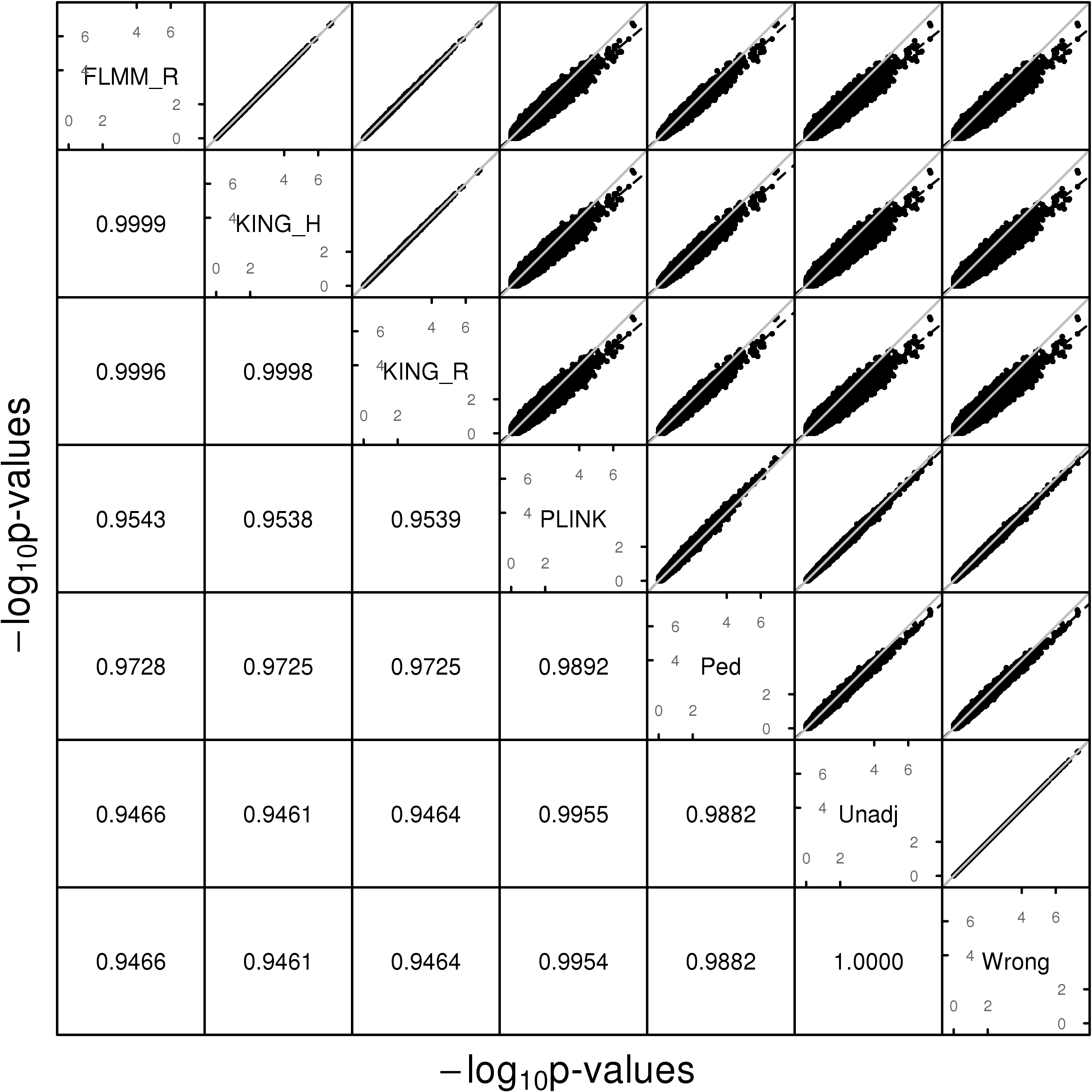

Supplement: Figure S10 — Comparison of −log10(p-values) obtained from FaST-LMM using alternative kinship estimates, real disease phenotypes. Plots above the diagonal show a comparison of –log10(p-values), with correlations between the –log10(p-values) indicated below the diagonal. The grey solid lines represents the line of equality; the black dashed lines the linear regression line of the variable on the y axis on the variable on the x axis. KING_H = KING homogeneous method, KING_R = KING robust method, Ped = theoretical kinship estimates based on pedigree information, FLMM_R = FaST-LMM's own realised relationship matrix, Unadj = unadjusted, Wrong = misspecified kinships, chosen to be inversely related to the true kinship value. (TIF) [file pgen.1004445.s010.tif]

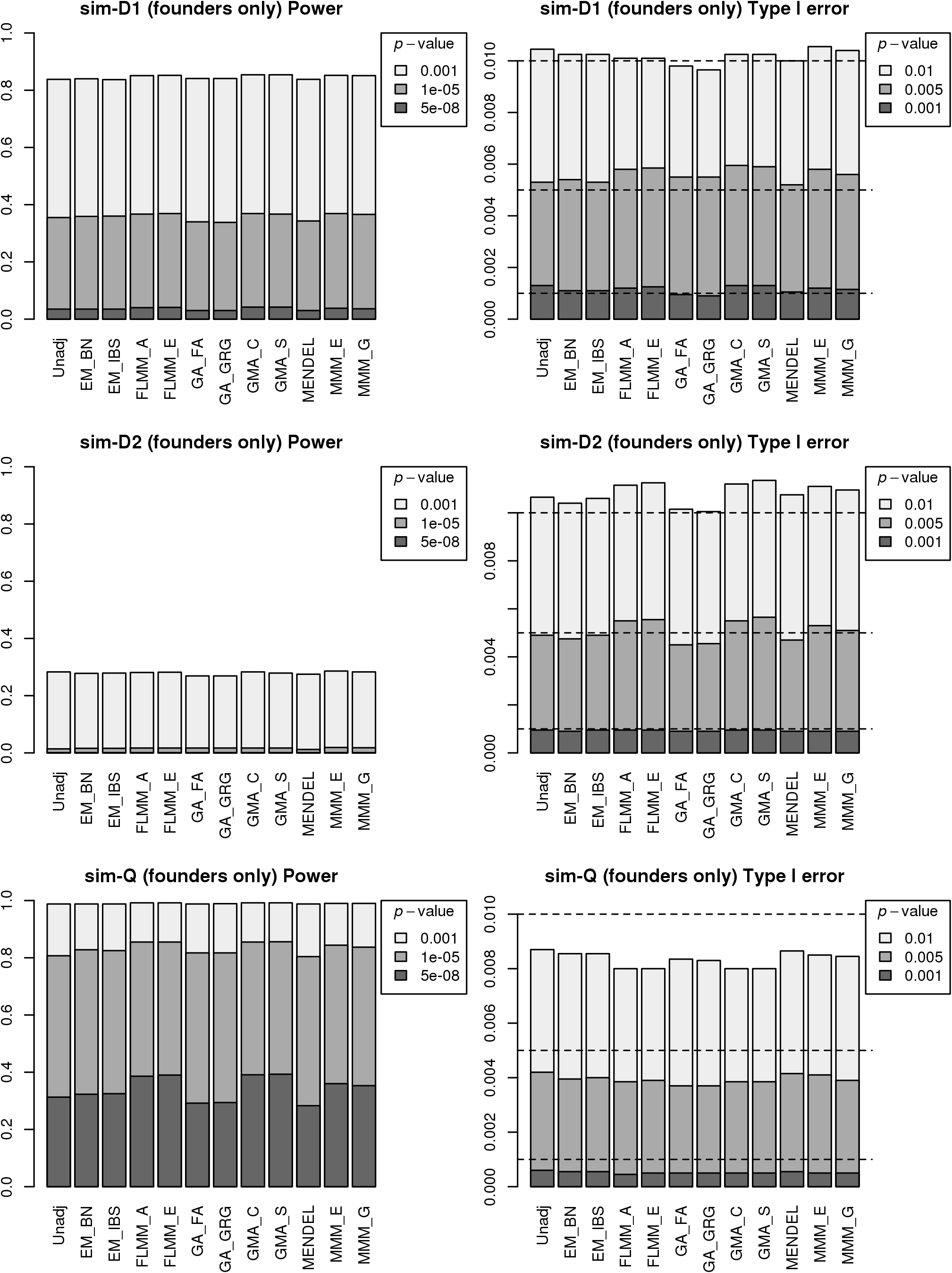

Supplement: Figure S11 — Power and type 1 error of different LMM methods applied to 462 Brazilian founders. Powers (left hand plots) are defined as the proportion of replicates (out of 1000) in which both simulated disease loci are detected, with ‘detection’ corresponding to any SNP within 40 kb of the simulated disease locus reaching the specified p-value threshold. Type 1 errors (right hand plots) are defined as the proportion of null SNPs (out of 20,000 = 20 null SNPs times 1000 simulation replicates) that reach the specified p-value threshold. Horizontal dashed lines indicate the target p-value thresholds (i.e. the expected type 1 error rates). (TIF) [file pgen.1004445.s011.tif]
