# Supplementary material for: Comparison of Methods to Account for Relatedness in Genome-Wide Association Studies with Family-Based Data
Source: PLoS Genet. 2014 Jul 17;10(7):e1004445. doi: 10.1371/journal.pgen.1004445 (PMC4102448; doi:10.1371/journal.pgen.1004445)
Supplement: Table S1 — Genomic control factors achieved in analysis of the real data, or a single replicate of the simulated data, when feeding externally estimated kinships into FaST-LMM. (PDF) [file pgen.1004445.s012.pdf]

**Table S1: Genomic control factors achieved in analysis of the real data, or a single replicate of the simulated data, when feeding externally estimated kinships into FaST-LMM**

| | Trait analysed | | |
|---|---|---|---|
| Kinship estimation method | Real | Simulated weak | Simulated quantitative |
| Unadjusted | 1.23 | 1.12 | 1.43 |
| Wrong[a] | 1.23 | 1.12 | 1.43 |
| FLMM-R[b] | 0.99 | 0.99 | 1.00 |
| KING (homogeneous) | 0.99 | 0.99 | 0.99 |
| KING (robust) | 0.99 | 0.99 | 1.00 |
| Theoretical (pedigree-based) kinships | 1.11 | 1.00 | 0.99 |
| PLINK | 1.18 | 1.12 | 1.42 |

[a] Wrong = misspecified kinships, chosen to be inversely related to the theoretical kinship value.

[b] FLMM-R = FaST-LMM's own realised relationship matrix
