# Supplementary material for: Comparison of Methods to Account for Relatedness in Genome-Wide Association Studies with Family-Based Data
Source: PLoS Genet. 2014 Jul 17;10(7):e1004445. doi: 10.1371/journal.pgen.1004445 (PMC4102448; doi:10.1371/journal.pgen.1004445)
Supplement: Table S2 — Computational speed and ease of use of various packages. (PDF) [file pgen.1004445.s013.pdf]

Table S2: Computational speed and ease of use of various packages

Package/method	Time taken to perform whole GWAS				Ease of use
	Data conversion from PLINK	Kinship calculation	Association analysis	Total	
EMMAX (BN)	8m19s	38s	14m40s	23m37s	Easy
EMMAX (IBS)	8m19s	43s	14m04s	23m06s	Easy
FaST-LMM (Approx)		(7-9s)	14m15s	14m15s	Easy
FaST-LMM (Exact)		(7-9s)	1h53m52s	1h53m52s	Easy
GEMMA (GMA_C)	-	2m49s	1h06m54s	1h09m43s	Easy
GEMMA (GMA_S)	-	2m48s	1h06m54s	1h09m42s	Easy
GenABEL (FASTA)	4m25s	11m44s	41m05s	57m14s	Requires familiarity with R
GenABEL (Grammar-Gamma)	4m25s	11m44s	25s	16m34s	Requires familiarity with R
Mendel (Theoretical kinships)	-	-	-	3m15s	Medium
Mendel (Estimated kinships)	-	-	-	2h27m02s	Medium
Mendel (Kinships estimated within estimated pedigree clusters)	1h34m4s	-	3m34s	1h37m38s	Medium
MMM (Approx)	18m01s	5m31s	29m33s	35m05s	Medium
MMM (Exact)	18m01s	5m06s	1h17m24s	1h40m31s	Medium
FBAT (Affecteds only)	25m	-	1h11m	1h36m	Medium
FBAT (Affecteds+unaffecteds)	25m	-	1h22m	1h47m	Medium
GTAM (implemented in MASTOR v0.3)	Varies	-	3h59m	3h59m +conversion time	File conversion fiddly
MASTOR	Varies	-	1h02m	1h02m +conversion time	File conversion fiddly
MQLS (1972 individuals)	14m	-	26m	40m	Medium
MQLS (3626 individuals)	25m	-	36m	1h01m	Medium
ROADTRIPS (1972 individuals)	Varies	-	15h36m	15h36m +conversion time	File conversion fiddly
ROADTRIPS (3626 individuals)	Varies	-	39h01m	39h01m +conversion time	File conversion fiddly
