# Supplementary material for: Comparison of Methods to Account for Relatedness in Genome-Wide Association Studies with Family-Based Data
Source: PLoS Genet. 2014 Jul 17;10(7):e1004445. doi: 10.1371/journal.pgen.1004445 (PMC4102448; doi:10.1371/journal.pgen.1004445)
Supplement: Table S3 — Concordance between top SNPs identified by different LMM methods when using 462 founder individuals. (PDF) [file pgen.1004445.s014.pdf]

Table S3: Concordance between top SNPs identified by different LMM methods when using 462 founder individuals

Trait	Method[a]	Mean (standard deviation) in 1000 replicates of proportion of top t SNPs within null and true regions that overlap with top t SNPs from EM_BN				
		$t = 5$	$t = 10$	$t = 15$	$t = 20$	$t = 25$
sim-D1	Unadjusted	0.978 (0.063)	0.993 (0.027)	0.991 (0.024)	0.989 (0.026)	0.988 (0.025)
	EM_IBS	0.998 (0.019)	0.997 (0.017)	0.996 (0.015)	0.996 (0.014)	0.996 (0.013)
	FLMM_A	0.981 (0.062)	0.978 (0.045)	0.969 (0.042)	0.968 (0.036)	0.965 (0.034)
	FLMM_E	0.981 (0.060)	0.977 (0.046)	0.969 (0.041)	0.968 (0.036)	0.965 (0.034)
	GA_FA	0.997 (0.024)	0.996 (0.021)	0.993 (0.021)	0.994 (0.017)	0.993 (0.017)
	GA_GRG	0.996 (0.027)	0.995 (0.022)	0.992 (0.022)	0.992 (0.020)	0.991 (0.019)
	GMA_C	0.980 (0.063)	0.976 (0.046)	0.967 (0.042)	0.965 (0.036)	0.963 (0.034)
	GMA_S	0.979 (0.063)	0.974 (0.048)	0.965 (0.042)	0.964 (0.036)	0.961 (0.034)
	Mendel	0.990 (0.044)	0.988 (0.033)	0.988 (0.026)	0.986 (0.024)	0.986 (0.023)
	MMM_E	0.993 (0.038)	0.990 (0.032)	0.987 (0.029)	0.986 (0.027)	0.986 (0.025)
	MMM_G	0.993 (0.038)	0.991 (0.031)	0.987 (0.031)	0.987 (0.027)	0.986 (0.025)
sim-D2	Unadjusted	0.960 (0.083)	0.984 (0.039)	0.985 (0.033)	0.984 (0.032)	0.986 (0.027)
	EM_IBS	0.995 (0.032)	0.996 (0.020)	0.993 (0.021)	0.994 (0.017)	0.995 (0.015)
	FLMM_A	0.959 (0.093)	0.958 (0.065)	0.956 (0.051)	0.956 (0.046)	0.958 (0.040)
	FLMM_E	0.960 (0.091)	0.958 (0.064)	0.956 (0.050)	0.956 (0.045)	0.958 (0.040)
	GA_FA	0.991 (0.043)	0.993 (0.029)	0.991 (0.025)	0.991 (0.022)	0.992 (0.019)
	GA_GRG	0.990 (0.047)	0.991 (0.032)	0.989 (0.029)	0.989 (0.025)	0.990 (0.021)
	GMA_C	0.958 (0.094)	0.955 (0.065)	0.954 (0.051)	0.953 (0.046)	0.956 (0.041)
	GMA_S	0.957 (0.095)	0.954 (0.066)	0.951 (0.051)	0.952 (0.046)	0.954 (0.040)
	Mendel	0.982 (0.063)	0.981 (0.041)	0.981 (0.035)	0.981 (0.030)	0.983 (0.027)
	MMM_E	0.983 (0.060)	0.984 (0.041)	0.982 (0.038)	0.983 (0.034)	0.983 (0.029)
	MMM_G	0.984 (0.058)	0.984 (0.042)	0.983 (0.038)	0.983 (0.034)	0.984 (0.029)
sim-Q	Unadjusted	0.997 (0.025)	0.995 (0.023)	0.991 (0.025)	0.991 (0.022)	0.990 (0.021)
	EM_IBS	0.999 (0.015)	0.998 (0.015)	0.998 (0.012)	0.997 (0.012)	0.996 (0.012)
	FLMM_A	0.988 (0.048)	0.983 (0.038)	0.979 (0.034)	0.975 (0.031)	0.974 (0.029)
	FLMM_E	0.988 (0.048)	0.983 (0.038)	0.979 (0.033)	0.976 (0.031)	0.974 (0.029)
	GA_FA	0.999 (0.015)	0.997 (0.017)	0.997 (0.014)	0.996 (0.014)	0.994 (0.015)
	GA_GRG	0.998 (0.018)	0.996 (0.020)	0.997 (0.015)	0.995 (0.016)	0.993 (0.016)
	GMA_C	0.987 (0.050)	0.981 (0.039)	0.978 (0.034)	0.974 (0.031)	0.972 (0.029)
	GMA_S	0.987 (0.050)	0.981 (0.039)	0.978 (0.034)	0.973 (0.032)	0.971 (0.029)
	Mendel	0.996 (0.028)	0.993 (0.026)	0.991 (0.023)	0.990 (0.021)	0.989 (0.020)
	MMM_E	0.995 (0.031)	0.993 (0.025)	0.993 (0.020)	0.991 (0.021)	0.991 (0.020)
	MMM_G	0.996 (0.027)	0.994 (0.024)	0.993 (0.020)	0.991 (0.021)	0.991 (0.020)

[a]See Table 2 for description of methods
